# Mechanism of ribosome shutdown by RsfS in *Staphylococcus aureus* revealed by integrative structural biology approach

**DOI:** 10.1038/s41467-020-15517-0

**Published:** 2020-04-03

**Authors:** Iskander Khusainov, Bulat Fatkhullin, Simone Pellegrino, Aydar Bikmullin, Wen-ti Liu, Azat Gabdulkhakov, Amr Al Shebel, Alexander Golubev, Denis Zeyer, Natalie Trachtmann, Georg A. Sprenger, Shamil Validov, Konstantin Usachev, Gulnara Yusupova, Marat Yusupov

**Affiliations:** 1 Laboratory of Structural Biology, Institute of Fundamental Medicine and Biology, Kazan Federal University, Kremlyovskaya Street 18, Kazan, 420008 Russia; 2 Department of Integrated Structural Biology, Institute of Genetics and Molecular and Cellular Biology, INSERM, U964, CNRS, UMR7104, University of Strasbourg, 1 rue Laurent Fries, F-67400 Illkirch, France; 3 Institute of Protein Research, Russian Academy of Sciences, Institutskaya 4, 142290 Puschino, Moscow Region Russian Federation; 4 NovAliX, BioParc, 850 bld Sebastien Brant, 67400 Illkirch, France; 5 Institute of Microbiology, University of Stuttgart, Allmandring 31, Stuttgart, 70569 Germany; 6 Present Address: Department of Molecular Sociology, Max Planck Institute of Biophysics, Max-von-Laue-Straße 3, 60438 Frankfurt am Main, Germany; 7 Present Address: Cambridge Institute for Medical Research, Department of Haematology, University of Cambridge, Cambridge, CB2 0XY UK

**Keywords:** Mechanism of action, Ribosomal proteins, Ribosome, Cryoelectron microscopy, X-ray crystallography

## Abstract

For the sake of energy preservation, bacteria, upon transition to stationary phase, tone down their protein synthesis. This process is favored by the reversible binding of small stress-induced proteins to the ribosome to prevent unnecessary translation. One example is the conserved bacterial ribosome silencing factor (RsfS) that binds to uL14 protein onto the large ribosomal subunit and prevents its association with the small subunit. Here we describe the binding mode of *Staphylococcus aureus* RsfS to the large ribosomal subunit and present a 3.2 Å resolution cryo-EM reconstruction of the 50S-RsfS complex together with the crystal structure of uL14-RsfS complex solved at 2.3 Å resolution. The understanding of the detailed landscape of RsfS-uL14 interactions within the ribosome shed light on the mechanism of ribosome shutdown in the human pathogen *S. aureus* and might deliver a novel target for pharmacological drug development and treatment of bacterial infections.

## Abstract (web summary)

Upon transition to stationary phase or upon stress, bacteria limit protein synthesis through small inhibitory proteins that bind the ribosome. Here the authors decipher the interaction mode of the bacterial ribosome silencing factor (RsfS) at atomic details to provide an in depth view of how it shutdowns ribosomes.

## Introduction

Upon transition to stationary phase or under stress conditions, bacteria abate their metabolism, largely by re-programming their protein synthesis apparatus through the interactions with a number of small proteins (reviewed in refs. ^1,2^). Recent high-resolution X-ray and cryo-electron microscopy (cryo-EM) structures revealed the mechanisms of action of some of these ribosome-bound proteins, including ribosome rescue factors SsrA-binding protein (SmpB), alternative ribosome rescue factor A (ArfA), alternative ribosome rescue factor B (ArfB)^3–9^, stringent factor RelA^10–12^, ribosome hibernation promoting factor (HPF), ribosome modulation factor (RMF), ribosome-associated inhibitor A (RaiA/YfiA)^13–15^, and ribosome splitting factor HflX^16^.

Most of the data available in the literature were obtained from ribosomal complexes of Gram-negative bacteria, such as *Escherichia coli* or *Thermus thermophilus*. However, only limited high-resolution structural investigations have been performed so far on ribosomes from gram-positive or/and pathogenic bacteria, such as *Bacillus subtilis*^17,18^, *Staphylococcus aureus*^19,20^, *Mycobacterium smegmatis*^21,22^, and *M. tuberculosis*^23^. The majority of these structures were devoted to the understanding of how bacteria can promote ribosome hibernation^24–28^, and indeed demonstrated the peculiar differences in the mechanism of function and proteins required for different bacteria. Notably, although *E. coli* and *T. thermophilus* promote hibernation by using distinct pathways^29,30^, the proteins from one bacteria can bind to the ribosome of another^14^.

Unlike HPF, the mechanism of stress response mediated by RsfS is conserved in bacteria (but it is also found in the mitochondria and chloroplasts); nonetheless, the knowledge of its mechanism of action and interaction with the ribosome is very limited. RsfS is a stationary phase protein that binds to the ribosomal protein uL14 on the large subunit (50S), and subsequently prevents its association with the small subunit (30S), thus tuning down translation during stress^31–33^. *E. coli rsfS* knocked-out cells show reduced adaptation during the transition from rich to poor media, with impaired viability during stationary phase^32^. Recently, a low-resolution (9 Å) cryo-EM reconstruction of RsfS bound to *M. tuberculosis* 50S ribosome revealed its binding region^22^; however, at this resolution, molecular details of the interaction interface and binding mode of RsfS with uL14 protein can not be described.

In this article, we show that RsfS increases the ratio of free ribosomal subunits under semi-dissociation conditions in *S. aureus* due to its anti-association activity. In order to gain further structural insights, we additionally obtained a 3.2 Å resolution cryo-EM structure of the 50S–RsfS complex reconstituted from 70S ribosomes under semi-dissociation conditions. Most importantly, we solved the crystal structure of the uL14–RsfS complex at a resolution of 2.3 Å and revealed the amino acids responsible for RsfS binding to ribosomal protein uL14 of the large subunit. Deciphering the interactions established by RsfS with the ribosome at high resolution provides an accurate perception about the general mechanism of the bacterial stress response, which has prominent clinical relevance in case of pathogens, such as *S. aureus*.

## Results

### Effect of RsfS on ribosomal subunits re-association

**Fig. 1:**
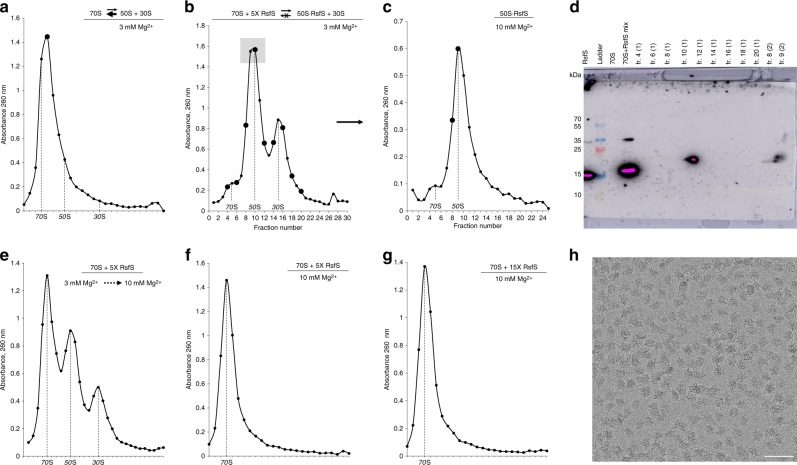
RsfS protein binds to the 50S particles and prevents ribosomal subunits association. **a** Sucrose gradient (SG) profile of the 70S at semi-dissociation conditions (3 mM Mg^2+^) with the equilibrium shifted toward the association. Bold marker indicates the fraction “70S” taken for the western blot analysis. **b**, **c** Purification of the 50S–RsfS complex for cryo-EM studies. SG profile of the 70S at 3 mM Mg^2+^ upon addition of 5X excess of RsfS (**b**), and subsequent centrifugation of the complex at 10 mM Mg^2+^ (**c**). The gray square indicates the fractions pooled for purification. Bold markers indicate the fractions taken for the western blot analysis. The peak of the 50S–RsfS complex (**c**) was taken for cryo-EM grid preparation and MS analysis. **d** Western blot analysis of the selected fractions. **e** SG profile of 70S + RsfS mixture that was incubated at 3 mM and spun at 10 mM Mg^2+^. **f**–**g** SG profiles of the 70S mixed 5× (**f**) or 15× (**g**) molar excess of RsfS, incubated and spun at 10 mM Mg^2+^. **h** Typical electron micrograph of the 50S–RsfS sample. Scale bar represents 50 nm. Source data for panels **a**–**c** and **e**–**g** are provided as a Source Data file.

To elucidate the binding mode of RsfS to the ribosome, we first determined the conditions at which the intact 70S ribosome shifts to a partially dissociated state. At these conditions, 70S ribosome and its subunits, 50S and 30S, would exist in a dynamic equilibrium, with a constant ratio between 70S and free subunits in solution. Since the association of bacterial ribosomes is most sensitive to the concentration of magnesium ions (Mg^2+^)^34^, we titrated Mg^2+^ while keeping the concentration of monovalent salts constant (Fig. 1a; Supplementary Fig. 1a). At 10 mM Mg^2+^, the 70S ribosome remains fully associated, whereas 2 mM causes full dissociation into individual subunits. Fine Mg^2+^ titration demonstrated that 3 mM and 2.5 mM Mg^2+^ lead to partial dissociation of the ribosome, with a majority of 70S in the first case and a majority of subunits in the latter (Fig. 1a; Supplementary Fig. 1a, middle panel).

The addition of 2–5 times molar excess of RsfS to the 70S, at 3 mM Mg^2+^, induced a remarkable shift of the equilibrium toward dissociation (Fig. 1b; Supplementary Fig. 1b, left and middle panels). The RsfS protein co-sedimented with the 50S subunit on a sucrose gradient, and in the absence of the 30S subunit, it remained bound to the 50S even at 10 mM Mg^2+^ (Fig. 1b–d). Conversely, increasing Mg^2+^ concentration from 3 to 10 mM in the 50S–RsfS + 30S mixture resulted in significant re-association of the ribosome back to 70S particles (Fig. 1e). At the same time, the integrity of the 70S at 10 mM Mg^2+^ was not affected by RsfS even using 15 times molar excess of the protein (Fig. 1f, g). These results demonstrate that RsfS binds to free 50S subunits and prevents its association with the 30S; however, RsfS is unable to trigger the 70S dissociation per se, and to prevent subunits re-association under conditions of elevated magnesium concentration.

### In vitro reconstitution of 50S–RsfS complex

Reconstitution of the 50S–RsfS complex was performed by mixing recombinant RsfS protein with 70S ribosome under conditions of partial dissociation followed by sucrose gradient-density centrifugation (Fig. 1b). This approach proved RsfS functional activity and guaranteed that the resulting peak of the 50S would correspond to the assembled complex rather than free 50S subunits. To minimize the presence of the remaining 70S and 30S particles in the sample, one additional step of sucrose gradient centrifugation was performed (Fig. 1c). In addition to western blot analysis (Fig. 1d), the presence of RsfS protein in the resulting 50S peak was confirmed by mass spectrometry (MS) (Supplementary Table 1). Several 30S proteins were present as minor contaminants, and others, e.g., bS1, uS10, uS14, uS15, uS18, and bS20, were absent. Among 50S proteins, bL7/L12, bL9, bL32, and bL34 were not detected by MS. In addition, the sample yet contained other contaminants: translation factor EF-Tu, pyruvate dehydrogenase complex E1 component alpha and beta subunits, and IgG-binding protein (these are coming from the 70S ribosomes purification procedure as described before^19^).

### Cryo-EM structure of *S. aureus* 50S–RsfS complex

**Fig. 2:**
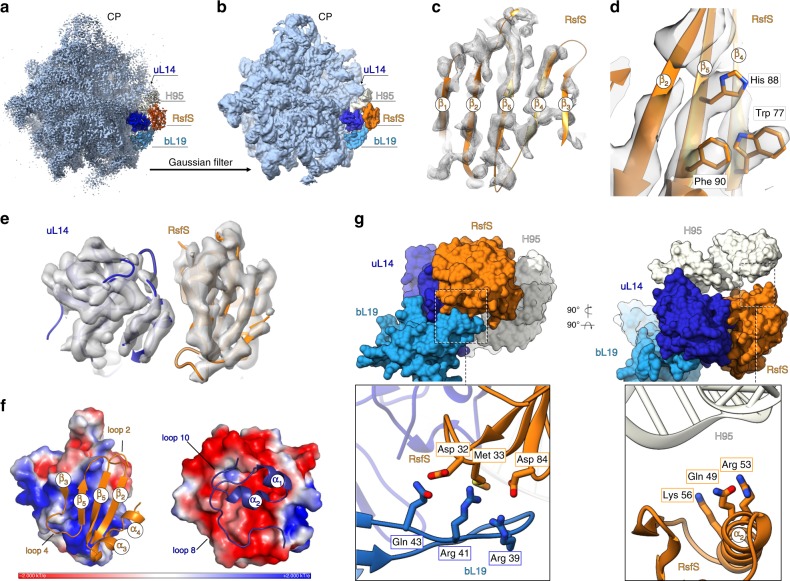
Cryo-EM reconstruction of the 50S–RsfS complex and model interpretation. **a** The 3.2 Å cryo-EM density map. Ribosomal protein uL14 is colored in dark blue, bL19 in bright blue, 23S rRNA Helix 95 in white, and RsfS in orange. CP central protuberance. **b** A low-pass filtered map of the 50S–RsfS complex (Gaussian filter with the width equal to 3.5 voxel size of the initial map) was used for the initial flexible fitting of the molecular model. **c**, **d** Density corresponding to the RsfS beta-sheet assembly and model is fitting. For representation reasons, a Gaussian filter with the width equal to one voxel size (outer mesh) was applied to the initial cryo-EM map (inner mesh). **e** The uL14–RsfS interaction interface as seen in the cryo-EM map (a Gaussian filter with the width equal to 1.5 voxel size applied). **f** Electrostatic potential of uL14 and RsfS (surface representation) calculated from the model; the structural elements involved in contacts formation are shown as ribbons and labeled. **g** The RsfS-binding cavity, including uL14, bL19, and H95. Molecules are shown in surface representation. Close-up sections demonstrate potential interacting residues of the proteins (represented as sticks).

Sample homogeneity and particles distribution were confirmed by negative staining electron microscopy on a Tecnai F20 microscope prior to data collection on a 300 kV Titan Krios microscope (Fig. 1h). The structure of the 50S–RsfS complex was resolved to an overall 3.2 Å resolution (Fig. 2a; Supplementary Fig. 2a). We did not identify any small ribosomal subunit proteins or non-ribosomal contaminants in the structure. As shown by local resolution estimation, the core region was mainly resolved to sub-3 Å resolution, with density for stacking nucleotides as well as amino acid side chains clearly resolved (Supplementary Fig. 2b, c). Peripheral regions, such as the 5S rRNA, as well as elements at the intersubunit interface and central protuberance, were distorted in the cryo-EM map probably due to extensive purification procedure in low Mg^2+^ concentration, which led to a significant drop of resolution in these areas (Fig. 2c). To build the model into these regions, we used a low-pass filtered map to fit the chains unambiguously (Fig. 2b–d).

The binding site of RsfS on the 50S ribosomal subunit is in overall agreement with the low-resolution cryo-EM reconstruction obtained for *Mycobacterium*^22^, but is not consistent with an earlier model exclusively inferred from genetic studies^32^. The complex formation is promoted by the interaction of the overall negatively charged beta-sheets of RsfS with the positively charged α-helices arrangement of uL14 (Fig. 2f). The RsfS-binding cavity also includes ribosomal protein bL19 and H95 of the 23S rRNA (Fig. 2g, f). More into details, RsfS potentially forms electrostatic interactions with residues Arg39, Arg41, Gln43 of bL19, while its additional anchoring point at the 50S may be mediated by positively charged residues (Gln49, Arg53, and Lys56) that interact with the negatively charged phosphate backbones of the 23S rRNA (Fig. 2g, f).

Structural comparison of the presented 50S–RsfS structure with the vacant *S. aureus* 70S structure^19^ shows that several elements near the intersubunit interface, such as H35, H38, H67, H69 of the 23S rRNA, and uL5, bL15, bL31 ribosomal proteins are likely destabilized by the absence of the 30S subunit, leading to a distorted or completely absent signal in the cryo-EM map (Supplementary Fig. 2d). RsfS-binding region on the 50S overlaps with intersubunit bridge B8, which is formed by uL14 and h14 of the 16S rRNA (Supplementary Fig. 2e). Structural analysis of the complex suggested a critical dual role for amino acid Arg97 of uL14, which mediates interaction with both A346 of the 16S rRNA and Tyr95 of RsfS, depending on the context in which it is involved. In both cases, the side chain of Arg97 interacts, through its amino groups, with the backbone of the respective partner, however, adopting two different conformations (Supplementary Fig. 2f).

### Crystal structure of *S. aureus* uL14–RsfS complex

**Fig. 3:**
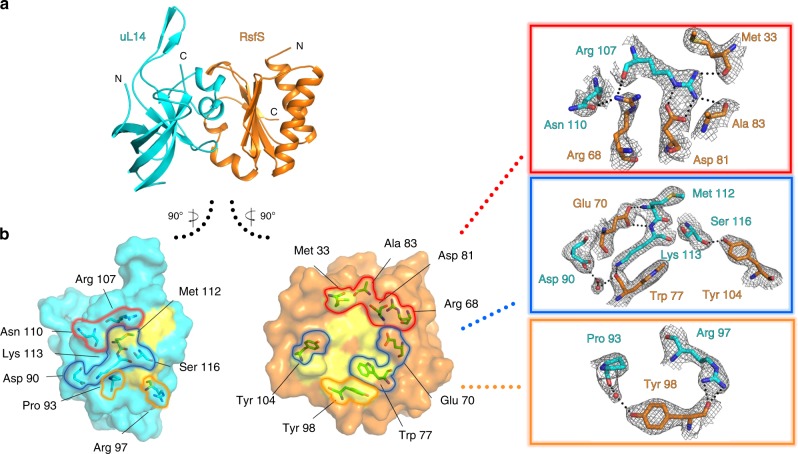
The crystal structure of uL14–RsfS complex and their interaction interface. **a** Atomic coordinates of one of the two uL14–RsfS heterodimers from the asymmetric unit. The Cα root-mean-square deviation (RMSD) between the two heterodimers was calculated to be 0.585 Å, suggesting that these two copies are essentially identical. The uL14 is shown in cyan, RsfS in gold; N and C stand for N-terminus and C-terminus of the proteins. **b** Amino acids of uL14 and RsfS proteins involved in contact formation. Hydrophobic regions are shown as yellow surfaces, contacting amino acids as sticks. Yellow, red, and blue frames show the H-bonds-forming amino acids form the respective regions marked on surface representation. H-bonds are shown as black dashed lines; the water molecules are shown as red ball; the crystallographic electron density is shown in mesh at 1.5 sigma value.

To interpret the binding of *S. aureus* RsfS to 50S ribosomal subunit in more details, we solved the crystal structure of RsfS bound to its interacting partner uL14 at 2.3 Å resolution (Fig. 3a). Expression and purification of individual proteins were prone to aggregation; thus, we co-expressed, purified, and crystallized *S. aureus* uL14–RsfS as an entire complex. The crystal structure demonstrates that the binding is mediated by a series of interactions, both electrostatic and hydrophobic (Fig. 3b). Importantly, the primary contacts between amino acids such as Arg97, Arg107, Lys113 of uL14, and Glu70, Asp81, Tyr98 of RsfS are conserved across bacteria. Notably, the Arg97 (uL14)–Tyr98 (RsfS) interaction observed in the cryo-EM structure is consistent with what we observe in the crystal structure. The complete list of interacting amino acids and their conservation is summarized in Supplementary Tables 2 and 3, and the full sequence alignments are shown in Supplementary Fig. 3a, b.

Overall, the uL14–RsfS interaction pattern seen in the crystal structure agrees well with the one identified in the cryo-EM structure. Pairwise structure alignment of each protein demonstrated their high structural similarity (C_α_ RMSD equals to 0.818 Å for 116 out of 122 pruned atom pairs of uL14 and 0.867 Å for 111 out of 112 pruned atom pairs for RsfS protein). However, the close-up view on the 3D alignment of the two obtained complexes onto uL14 protein shows that helices α2 and α3 of RsfS do not overlap well (Supplementary Fig. 4a). We speculate that such differences at the solvent facing side of RsfS are probably due to the fact that this region is, to some extent, flexible in solution, but stabilized in crystals. Nevertheless, the quality of the cryo-EM reconstruction allowed to assign bulky side chains (i.e., Tyr36, Trp77, His88, Phe90) and ensured reliable flexible fitting of RsfS (Fig. 2c–e).

## Discussion

In this work, we uncover the binding mode and describe the detailed interactions that the stress protein RsfS establishes with the 50S ribosomal subunit in *S. aureus* using cryo-electron microscopy and X-ray crystallography. Experiments on 50S–RsfS complex formation under different magnesium concentrations proved that RsfS does not promote subunits dissociation, but rather functions as an anti-association factor. This means that the protein may prevent the last step of the translation initiation process when the large ribosomal subunit joins the 30S initiation complex. The binding site of RsfS does not overlap with any translation factors, which excludes the possibility of a competition mechanism. However, it does not overlap with any known antibiotic’s binding site either; thus, most probably, this stress factor can not protect bacteria under antibiotics pressure. This grants to RsfS the role of a fine modulator of protein synthesis in cells upon entry in stationary phase, which acts independently from other translation regulators such as translation factors and other stress-induced proteins, such as HPF, YfiA, RMF, or EttA^2^. Therefore, a proper activity of this highly conserved bacterial protein may be necessary for the survival of bacterial cells under unfavorable conditions.

Our cryo-EM structure, further supported by mass-spectrometry data, shows that RsfS indeed binds to ribosomal protein uL14 as previously suggested^22,32^. However, the higher resolution of our map, together with the crystal structure of the uL14–RsfS complex, revealed the critical amino acids required for this interaction to occur (Fig. 3a, b). Multiple sequence alignment of uL14 and RsfS proteins from 16 different species revealed that the amino acids involved in binding of RsfS to the ribosome are highly conserved (Supplementary Fig. 3a, b). The first glimpse on RsfS effect on 50S ribosome during stress was obtained from mutagenesis studies of highly conserved amino acids in *E. coli* uL14^32^. This work led the authors to identify RsfS as a binding partner of uL14^32^, and to predict, although with high approximation, the interactions that could have been involved. Mutations T97A and K114A were found to be the most critical, while R98A had less importance, and S117A did not affect uL14–RsfS complex stability^32^. In *S. aureus*, these amino acids correspond to Thr96, Lys113, Arg97, and Ser116, respectively (Supplementary Fig. 3a). In our crystal structure, Thr96 and Lys113 of uL14 indeed interact with RsfS; however, they form only a few H-bonds, thus unlikely representing the most crucial interacting interface. Interestingly, our structures suggest that Arg97 undergoes a structural rearrangement of its side chain to accommodate either RsfS or the 16S rRNA (when RsfS is not present), becoming a determinant of the transition to and from stress conditions. Notably, Arg97 is conserved in evolutionary distant species (Supplementary Fig. 3a), and its side chain mediates a direct interaction with the backbone of Tyr98 of RsfS or the phosphate backbone of A346 of the 16S rRNA (Supplementary Fig. 2f). We thus suggest that such mechanism of binding partner determination is sequence-independent, and it might be universal across bacteria. Furthermore, in agreement with the genetic data produced in *E. coli*^32^, we show that in *S. aureus* Ser116 interacts with RsfS via its backbone; therefore, replacement of Ser116 side chain should not have a direct effect on complex formation and stability. A previous low-resolution cryo-EM structure in *M. tuberculosis* allowed to visualize RsfS on the 50S more accurately^22^. In this work, the authors found that recombinant RsfS protein from *M. tuberculosis* formed a homodimer in solution; in addition, the replacement of the conserved Glu74 by alanine granted increased solubility, but significantly decreased its affinity to 50S. In *S. aureus*, this residue corresponds to Glu70, which is a prerequisite for binding to uL14, well in agreement with *M. tuberculosis* data. However, in our experiments, we co-expressed uL14 and RsfS, and we did not observe RsfS homodimer neither in solution, as seen from the gel filtration profile (Supplementary Fig. 5a, b), nor in the crystal structure. Thus, we suggest that *S. aureus* wild-type RsfS does not tend to form homodimers in the presence of uL14 protein.

Conservation of uL14 and RsfS amino acid sequences from different bacteria (Supplementary Fig. 3a, b) is translated in high similarity also at the structural level. The structure of *S. aureus* uL14 within the crystallographic complex with RsfS was almost identical to the individually crystallized uL14 protein from *Geobacillus stearothermophilus*^35^, as well as to uL14 within the ribosome of *S. aureus*, *T. thermophilus*, and *M. tuberculosis* (Supplementary Fig. 4b). *S. aureus* RsfS from uL14–RsfS complex shows high structural similarity (C_α_ RMSD of 0.877 Å for 93 out of 112 pruned atom pairs) to the individually crystallized RsfS from *M. tuberculosis*^22^ with the exception of the loop 4 and α3-helix in *Mycobacterium* that separates into two individual helices, α3-α4 in *S. aureus* (Supplementary Fig. 4c). Superposition of uL14–RsfS complexes in our cryo-EM and crystal structure shows that the structure of uL14 protein is identical in both complexes, regardless of whether it is part of the ribosome or individual protein in solution, whereas RsfS adopts a less compact conformation in the cryo-EM structure (Supplementary Fig. 4).

**Fig. 4:**
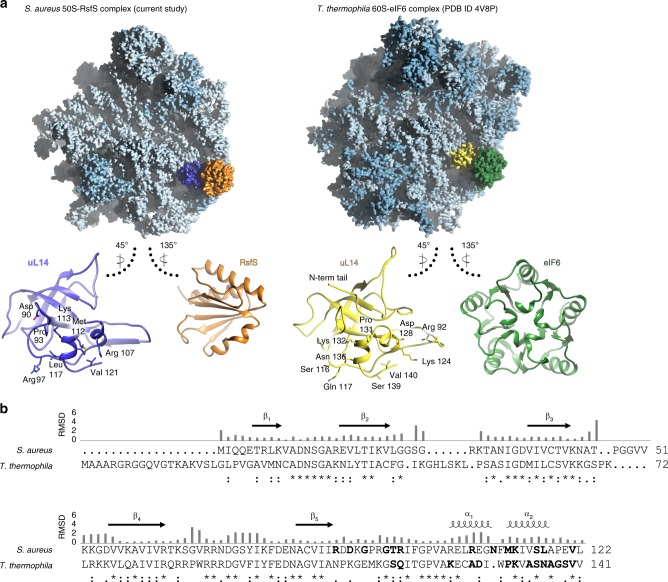
Eukaryotic initiation factor eIF6 shares a similar binding site on universal ribosomal protein uL14, but has a different structure. **a** Localization of RsfS and eIF6 proteins on *S. aureus* 50S and *Tetrahymena thermophila* 60S, respectively. For a visual representation, the maps were simulated from the atomic coordinates of the models. Ribosomal RNAs are colored in light blue, ribosomal proteins in blue, *S. aureus* uL14 in dark blue, RsfS in orange, *T. thermophila* uL14 in yellow, and eIF6 in green. Individual proteins are shown in ribbon to represent their interaction interface, which displays the structural similarity of uL14 and the dissimilarity of the RsfS and eIF6 proteins in the two organisms. Residues of uL14 involved in the interaction with RsfS/eIF6 are shown as sticks, and labeled accordingly. **b** Pairwise sequence alignment of uL14 from *S. aureus* and *T. thermophila* demonstrates their high sequence similarity. Secondary structure elements are shown above the alignment. Residues interacting with RsfS (or eIF6) are highlighted in bold. The RMSD (given in Å) of 3D structure alignment is shown as bars above the sequence. An asterisk indicates a single, fully conserved residue (identity), colon indicates conservation between amino acids with substantial chemical similarity, while dot indicates conservation between amino acids with poor chemical similarity.

Notably, RsfS occupies the same binding site as initiation factor 6 (eIF6) in eukaryotes^36^ (Fig. 4a), which possesses ribosome anti-association activity to prevent the formation of cytoplasmic 80S ribosome^37,38^. In addition, eIF6 (TIF6 in yeast) is involved in 60S biogenesis and its nuclear export^39,40^. Similarly, RsfS homologs in human mitochondria and plant plastids (C7orf30/MALSU1 and Iojap, respectively) appear to be involved predominantly in ribosome biogenesis^10,33,41–43^, although some experiments also demonstrated their activity to downregulate translation^32^. To date, there is no evidence that RsfS may participate in ribosome biogenesis in bacteria, as well as the mechanism of dissociation of RsfS from the 50S remains elusive. Considering that RsfS is firmly bound to the 50S when the latter becomes available, it is plausible that a source of energy and additional factors might be required for its dissociation, as in the case of eIF6 release from the 60S^44,45^. It is known that in *S. aureus*, HPF dissociation from the ribosome is based on GTP availability and performed by HflX GTPase^46^. We can speculate that an HflX-like mechanism may also be valid for RsfS: upon transition to favorable conditions, accumulation of GTP might trigger GTPase(s) to remove RsfS from the 50S. However, based on the available data, it is more likely that, despite the conservation of uL14 in all kingdoms of life and RsfS in bacteria, mitochondria, and chloroplasts, in addition to an overlapping binding site with eIF6, a functional divergence occurred between these proteins during evolution.

In conclusion, our data provide a detailed structural interpretation of one of the mechanisms of ribosome shutdown during stress response in *Staphylococcus aureus*. Despite this mechanism is conserved in bacteria, more biochemical data and perturbation experiments will be required to fully understand the essentiality of RsfS for the survival of *S. aureus*. The crystal structure and the detailed comparative analyses we provide represent a groundwork for such studies and will facilitate the design of mutagenesis experiments in *S. aureus* and for analysis of stress survival rates upon disruption of RsfS/uL14 interface. All this will shed new lights into the specificity of these interactions in evolutionary distinct bacteria and lead to the design of *S. aureus*-specific compounds aimed to decrease the survival of this multi-drug-resistant human pathogen.

## Methods

### Ribosomes purification

The vacant 70S ribosomes were purified form the RN6390 strain of *Staphylococcus aureus* grown at 37 °C (180 rpm) to OD_600_ = 1.0. Cells were lysed in buffer A (20 mM HEPES–KOH pH 7.5, 100 mM NH_4_Cl, 21 mM Mg-acetate, 1 mM EDTA, 1 mM DTT) containing 1× protease inhibitors solution (Roche), and lysostaphin (Sigma-Aldrich) 0.5 mg per gram of cells. After centrifugation 30,000 × *g* for 90 min, cell lysate was supplemented with 2.8% of PEG 20,000 (Hampton Research) spun 10,000 × *g* for 5 min. The PEG 20,000 concentration was increased to 4.2% in the supernatant. After centrifugation 10,000 × *g* for 10 min, the ribosome pellet was dissolved in buffer A and passed through a sucrose cushion (10 mM HEPES–KOH pH 7.5, 500 mM KCl, 25 mM Mg-acetate, 1.1 M sucrose, 0.5 mM EDTA, 1 mM DTT) at 100,000 × *g* for 15 h using a Beckman Type 45 Ti rotor. The pellet was resuspended in 10 mM HEPES–KOH pH 7.5, 100 mM KCl, 10 mM Mg-acetate, 0.5 mM EDTA, 1 mM DTT, and the ribosomes were separated on 7–30% sucrose gradients equilibrated in the same buffer and spun at 38,694 × *g* for 15.5 h using a Beckman SW28 rotor. The fractions corresponding to 70S particles were pooled, the concentration of Mg-acetate was adjusted to 25 mM, and PEG 20,000 was added to a final concentration of 4.5% w/v. Ribosomes were pelleted by centrifugation at 20,000 × *g* for 12 min, the pellet was gently dissolved in buffer G (10 mM HEPES–KOH pH 7.5, 50 mM KCl, 10 mM NH_4_Cl, 10 mM Mg-acetate, 1 mM DTT).

### Sucrose gradient analysis

For magnesium titration experiments (Fig. 1a; Supplementary Fig. 1a) 70S ribosomes were dialyzed in buffer H_10_K_50_N_10_ (10 mM HEPES–KOH pH 7.5 (at 25 °C), 50 mM KCl, 10 mM NH_4_Cl) containing the respective Mg^2+^ concentration for at least 3 h, and loaded onto sucrose gradients prepared in the same buffer. For RsfS-binding experiments, 70S ribosomes were incubated with 1×, 2×, 5×, or 15× molar excess of RsfS protein in buffer H_10_K_50_N_10_M_3_ (M_3_ stands for 3 mM Mg-acetate). The final buffer conditions were adjusted to H_10_K_50_N_10_M_3_, the mixture was incubated for 30 min at 37 °C, and loaded onto sucrose gradients prepared in either H_10_K_50_N_10_M_3_ buffer (Fig. 1b; Supplementary Fig. 1b) or H_10_K_50_N_10_M_10_ buffer (Fig. 1e). To evaluate RsfS dissociation activity (Fig. 1f, g), 70S + RsfS mixture was incubated and applied similarly to sucrose gradients, but keeping always Mg^2+^ concentration at 10 mM. All sucrose gradients performed in this study were prepared with a linear 0–30% gradient of sucrose in buffer H_10_K_50_N_10_ supplemented with different concentrations of Mg-acetate (2 mM, 2.5 mM, 3 mM, or 10 mM). All centrifugations were performed using a Beckman SW41 rotor running at 53,000×*g* for 12.5 h at 4 °C. Fractions of 0.4 mL were collected and measured using Nanodrop 2000 (Thermo Scientific™) at 260 nm wavelength. Three different preparations of the 70S sample were used in the study.

### Purification of 50S–RsfS complex

*S. aureus* RsfS with a six-histidines tag at the N-terminus was cloned into pET28 plasmid and recombinantly expressed in *E. coli* BL21 (DE3) pLysS cells. Cells were grown at 37 °C until an OD_600_ of 1.0, and protein expression was induced by the addition of 0.5 mM IPTG at 30 °C for 5 h. Collected cells were resuspended in lysis buffer (20 mM Tris-HCl, 300 mM NaCl, 10 mM imidazole, pH 7.5), and disrupted by sonication. The resulting lysate was cleared by 30 min centrifugation at 30,000×*g* and then passed through a Ni-NTA resin (Qiagen) equilibrated in lysis buffer, washed with the same buffer containing 20 mM imidazole and eluted with lysis buffer supplemented with 250 mM imidazole. Eluted fractions were precipitated by adding ammonium sulfate salt powder until 80% of saturation and stirring for 1 h at 4 °C. Prior to complex formation with the ribosome, RsfS precipitate was re-solubilized in H_10_K_50_N_10_ buffer and purified on Superdex 75 10/300 (GE-Healthcare) to remove aggregates. The peak fraction was collected for 50S–RsfS complex preparation.

To assemble the 50S–RsfS complex, vacant *S. aureus* 70S ribosomes were dialyzed in semi-dissociation buffer H_10_K_50_N_10_M_3_ and mixed with five times molar excess of RsfS protein. The mixture was incubated at 37 °C for 30 min and loaded onto sucrose gradients prepared in the same buffer (Fig. 1b). The fractions containing 50S–RsfS were buffer exchanged to H_10_K_50_N_10_M_10_. Afterward, the sample was concentrated using a Centricon with a molecular weight cutoff (MWCO) of 30 kDa, and finally layered onto another sucrose gradient prepared in the same buffer (Fig. 1c). Sucrose was removed by buffer exchange, and the sample was concentrated using a Centricon MWCO 30 kDa. Peak fractions were used for MS analysis, negative staining and cryo-EM grids preparation.

### Western blot analysis

From each selected sucrose gradient fraction (Fig. 1b,c), 10 µL were mixed with equal volume of 2× loading buffer (120 mM Tris-HCl pH 7.5 (25 °C), 40% glycerol, 0.09% bromophenol blue, 20 mM DTT). As control samples, we used RsfS protein (0.1 µg), 70S ribosome (1.1 µg) fraction from sucrose gradient at 3 mM Mg^2+^ (Fig. 1a) and 70S + 5X RsfS mixture (containing 1.5 µg 70S and 0.35 µg RsfS) before centrifugation in sucrose gradient. Proteins were separated on 15% polyacrylamide gel according to Leammli electrophoresis protocol^47^. The proteins were transferred to a 0.45 µm nitrocellulose membrane at 10–11 V for 45 min at room temperature using the Trans-Blot Semi-Dry Transfer apparatus (BioRad). After incubation with 5% nonfat milk in TBST (10 mM Tris, pH 8.0 150 mM NaCl, 0.5% Tween 20) for 60 min at 37 °C, the membrane was washed once with TBST and incubated with monoclonal anti-polyhistidine-peroxidase clone HIS-1 antibody (Sigma-Aldrich) at 1:2000 ratio at 37 °C for 1 h. Membranes were washed four times for 10 min and developed with Pierce™ ECL western blotting substrate, and detected by Thermo SuperSignal™ West Femto Maximum Sensitivity Substrate and the ECL system (Amer Biosciences) according to the manufacturer’s protocols.

### Negative staining

For negative stain, 2.5 µL of the sample was placed on the top of a glow-discharged grid coated with continuous carbon film and incubated at room temperature for 10 s to allow the proper amount of particles to adsorb. The solution was then blotted away from the grid with a filter paper. A 2.5 µL droplet of uranyl acetate solution was afterward applied on the grid for 1 min to stain the particles. Excess of uranyl acetate solution was blotted away, and the grid was left to air dry before imaging. A Tecnai F20 electron microscope equipped with Falcon II detector operated at 200 kV was used to acquire a negative stain image at room temperature, with a pixel size of 1.69 Å/pix and a total electron dose of ~50 electrons/Å^2^.

### Cryo-EM data collection

Cryo-EM grids were prepared with the Vitrobot (FEI Company) equilibrated at 4 °C and 100% relative humidity. Four microliters of the sample were applied onto the Quantifoil R2/2 holey carbon grid, which had been coated with thin carbon film and glow-discharged. After 30 s wait and subsequent blotting, the grids were flash-frozen in liquid ethane. Data collection was performed on a Titan Krios electron microscope (FEI Company) at 300 kV, using the EPU software (FEI Company) for automated data acquisition. Data were collected with a defocus of −1.2 to −3 μm at a magnification of 96,000×, giving a nominal pixel size of 0.858 Å/px. The micrographs (1856) were recorded on Falcon III direct electron detector (FEI Company) as movie stacks. The exposure time for each movie stack was ~43 s, corresponding to an electron dose of ~0.6 electrons/Å^2^ fractionated into 41 frames (~1.02 s/frame). Drift, gain corrections, and dose-weighting were performed with MotionCor2^48^ using all frames on 1 × 1 patches.

### Data processing and map calculation

All the data processing has been performed using the Scipion suite^49^. The contrast transfer function (CTF) was calculated from motion-corrected and non-dose-weighted images using GCTF^50^. After removing images having poor CTF quality (leaving a total of 1758 micrographs), ribosome particles were picked from motion-corrected and dose-weighted images using Xmipp as implemented in Scipion^51^. After extraction and fivefold binning (4.29 Å/px) of 172,729 particles, reference-free 2D classification using RELION 2.1^52^ was performed with 120 classes. After the removal of non-ribosomal particles, the remaining 163,765 particles were subjected to three-dimensional (3D) classification using ten classes. As an initial reference, low-pass filtered to 60 Å, we used the map of the *Mycobacterium tuberculosis* 50S ribosome in complex with RsfS (EMDB 6177)^22^. After classification, 127,598 selected particles were extracted, unbinned to their initial pixel size, and subjected to 3D auto-refinement using the same reference as for the 3D classification job. Post processing of the resulting map using automatic B factor estimation as a sharpening procedure allowed to obtain a reconstruction estimated to be at a resolution of 3.26 Å using a FSC = 0.143 gold-standard threshold. To validate the resulting map and exclude any reference bias at the level of Rsf, we additionally generated an initial model using the set of particles derived from 2D classification, as implemented in Relion 3.0^53^, which uses a stochastic gradient-descent (SGD) algorithm to generate an ab initio map, without need for an external reference. The output was used as a reference to run 3D classification and 3D refinement jobs within Relion 3.0. The generated maps were analyzed and showed clearly no differences, revealing in all cases a density in the region where RsfS is suggested to bind (Supplementary Fig. 6). We additionally performed 3D classification and 3D refinement jobs using a model of the vacant *S. aureus* 50S (EMD-7870), and after visual inspection, the map displayed density for Rsf as for the two previous ones.

Afterward, we extracted the particles used for 3D refinement from all the 40 movie-frames using Xmipp^51^ and performed movie-refinement and particle polishing jobs in RELION 2.1^52^. The final resolution of the generated map at this step was 3.16 Å, at a gold-standard FSC = 0.143, after post processing. The output map of the 3D refinement job was used to create two masks: one around the region where RsfS should be located, based on the previous low-resolution structure^22^, and the other comprising the 50S ribosomal subunit excluding this region. The latter mask was used for signal subtraction job in RELION 2.1^52^, and the resulting subtracted particles were subjected to two consecutive cycles of focused 3D classification on the RsfS region, without image alignment and using the previously obtained 3D refined map from the shiny particles, low-pass filtered to 40 Å. We used five and two classes, respectively, to sort sample heterogeneity. The final full-size images of 83,885 particles were processed by 3D auto-refine, and the resulting overall average resolution of the map was 3.23 Å, at a gold-standard FSC = 0.143, after post processing. All maps were sharpened using auto-bfac option in RELION 2.1^52^. Local resolution was estimated using Relion 3.0^53^.

### Model building and validation

For model building, 50S subunit was extracted from the *S. aureus* vacant 70S ribosome model (PBD 5LI0 [10.2210/pdb5LI0/pdb])^19^ and rigid body fitted into Gaussian filtered cryo-EM density map of 50S–RsfS complex (Fig. 2b). uL14 from this model was replaced by the crystal structure of uL14–RsfS complex (this study), and flexible regions of the model that could not fit into the density were deleted. The initial coarse fitting of the flexible elements at the intersubunit interface and periphery, as well as RsfS, into the density, was performed using the NAMDinator web service^54^, which implements the algorithms of molecular dynamics flexible fitting (MDFF^55^), and phenix.real_space_refinement^56^. The default parameters (start temperature = 298 K; G-force scaling factor = 0.3; minimization steps = 2000; simulation steps = 20,000) were used for flexible fitting, while real-space refinement was performed separately in phenix^56^, including simulated annealing (starting temperature = 800 K; cool rate = 100 K) and global minimization. Model and map were visually inspected in Coot^57^. The secondary structure elements and bulky aromatic amino acids were first docked during refinement of the RsfS model (Fig. 2c–e). The densities that can be attributed to solvent molecules have been interpreted as Mg^2+^ ions, while in order to model K^+^ ions the structure was aligned to *T. thermophilus* 70S model with experimentally assigned K^+^ ions^58^. Final minimization of coordinates (global minimization with hydrogen atoms) was carried out in Phenix real-space refinement at default parameters^55^. For model validation, we used the MolProbity webserver^59^ and model-to-map correlation statistics from Phenix. Data and refinement statistics are summarized in Table 1.

**Table 1:** Cryo-EM data collection, refinement, and validation statistics.

	50S–RsfS (EMD-10212) (PDB 6SJ6)
*Data collection and processing*	
Magnification	96,000×
Voltage (kV)	300
Electron exposure (e–/Å^2^)	24.6
Defocus range (μm)	−1.2 to −3
Pixel size (Å)	0.858
Symmetry imposed	C1
Initial particle images (no.)	172,729
Final particle images (no.)	83,885
Map resolution (Å)	3.2
FSC threshold	0.143
Map resolution range (Å)	3.0–15.0
*Refinement*	
Initial model used (PDB code)	5LI0
Model resolution (Å)	3.8
FSC threshold	
Model resolution range (Å)	3.5 – 20.0
Map sharpening *B* factor (Å^2^)	−63.2
Model composition	
Non-hydrogen atoms	78,315
Protein residues	2611
Nucleotides	2697
Ligands	59
*B* factors (Å^2^)	
Protein	36.12
RNA	56.39
Ligand	20.01
R.m.s. deviations	
Bond lengths (Å)	0.009
Bond angles (°)	1.119
*Validation*	
MolProbity score	1.99
Clashscore	4.33
Poor rotamers (%)	0.55
Ramachandran plot	
Favored (%)	82.18
Allowed (%)	17.67
Disallowed (%)	0.16

### Expression and purification of uL14–RsfS complex

The uL14 and RsfS of *S. aureus* were cloned into a modified pACYCDuet-1 plasmid with a six-histidine tag fused at the N-terminus of uL14 and co-expressed in *E. coli* BL21star(DE3) cells. Protein expression was induced by adding 0.5 mM IPTG to the cell culture when an OD_600_ of 0.6 was reached. The cells were harvested after 4 h of induction at 37 °C. Upon cell disruption by sonication in buffer A (20 mM Tris-HCl, 500 mM NH_4_Cl, pH 8.0) supplemented with phenylmethylsulfonyl fluoride (PMSF), cell lysate was cleared by 30 min centrifugation at 30,000×*g* followed by 1 h ultracentrifugation at 100,000×*g*. The heterodimer uL14–RsfS was further purified by Ni-NTA chromatography and eluted in a buffer A containing 300 mM imidazole. As final step of purification, the protein complex was applied to a size-exclusion chromatography HiLoad 16/600 Superdex 75 prep-grade column equilibrated in Buffer B (50 mM Na_2_HPO_4_/NaH_2_PO_4_, pH 7.0 and 0.2 M NaCl (Supplementary Fig. 5a). Complex formation and purity was verified by SDS-PAGE (Supplementary Fig. 5b).

### Crystallization, data collection, and structure determination

The uL14–RsfS complex was crystallized using the hanging drop vapor diffusion method. The drops were prepared by mixing 1.2 μL of protein solution with 1.2 μL reservoir solution (0.1 M MES pH 6.0, 0.2 M lithium sulfate, 20% PEG 4000). Drops were equilibrated against 250 μL reservoir solution at 20 °C. Crystals appeared after 5–7 days, and were cryo-protected directly before X-ray data collection by a custom-made cryoprotectant solution (0.17 M ammonium sulfate, 25.5% PEG 8000, 15% glycerol). Preliminary X-ray data (at 4.2 Å resolution) at 100 K were collected using a Cu K-alpha radiation from a PhotonJet-S microfocus sealed tube X-ray generator (Rigaku XtaLAB Synergy-S, Kazan Federal University) equipped with µ-CMF optics (Rigaku Oxford Diffraction) and an HyPix-6000HE detector. The high-resolution diffraction data set was collected from a single crystal on ID30B beamline at the European Synchrotron Radiation Facility (ESRF, France). Diffraction data were collected using a wavelength of 0.9762 Å on a PILATUS 6 M detector with parameters experimentally optimized based on crystal mosaicity. Data were processed with XDS program package^60^. The structure was solved by molecular replacement using Phaser from the Phenix package^61^. Protein uL14 from *S. aureus*, (PDB 5ND8 [10.2210/pdb5nd8/pdb]) and protein Q9KD89 from *Bacillus halodurans*, (PDB 2O5A [10.2210/pdb2O5A/pdb]) were used as starting models. The initially obtained model was refined using phenix.refine^62^, followed by iterative manual building in COOT (Emsley et al.^57^) and refinement cycles in phenix.refine. Refined model contained 94.37% Ramachandran favored, 5.63% Ramachandran allowed and 0% Ramachandran outliers. Data and refinement statistics are summarized in Table 2. All figures were prepared with Chimera and Pymol^63,64^.

**Table 2:** Data collection and refinement statistics (molecular replacement).

	Crystal 1
*Data collection*	
Space group	*P*2_1_2_1_2_1_
Cell dimensions	
* a*, *b*, *c* (Å)	37.11, 107.60, 121.22
α, β, γ (°)	90.00, 90.00, 90.00
Resolution (Å)	42.20–2.27 (2.35–2.27)*
*R*_sym_ or *R*_merge_	0.05 (1.53)
*I*/σ*I*	14.53 (0.9)
Completeness (%)	98.95 (99.17)
Redundancy	4.48 (4.39)
*Refinement*	
Resolution (Å)	42.2–2.27
No. of reflections	25,247
*R*_work_/*R*_free_	0.23/0.27
No. of atoms	3709
Protein	3672
Ligand/ion	21
Water	16
*B* factors	
Protein	88.25
Ligand/ion	98.95
Water	66.82
R.m.s. deviations	
Bond lengths (Å)	0.01
Bond angles (°)	1.13

### Mass spectrometry

The 50S–RsfS sample was reduced, alkylated, and digested with trypsin at 37 °C overnight. Extracted peptides were then analyzed using an Ultimate 3000 nano-RSLC (Thermo Scientific, San Jose California) coupled in line with an Orbitrap ELITE (Thermo Scientific, San Jose California). Briefly, peptides were separated on a C18 nanocolumn with a linear gradient of acetonitrile and analyzed in a Top 20 CID (Collision-induced dissociation) data-dependent mass spectrometry. Data were processed by database searching against *Staphylococcus aureus* Uniprot Proteome database using Proteome Discoverer 2.1 software (Thermo Fisher Scientific). Precursor and fragment mass tolerance were set at 7 ppm and 0.6 Da, respectively. Trypsin was set as an enzyme, and up to two missed cleavages were allowed. Oxidation (M), N-term acetylation were set as variable modification and carbamidomethylation (C) as a fixed modification. Proteins were identified with a minimum of two unique peptides, and were filtered with false discovery rate <1%.

### Reporting summary

Further information on research design is available in the Nature Research Reporting Summary linked to this article.

## Supplementary information

SUPPLEMENTARY INFORMATION

Peer Review

Reporting Summary

## Data Availability

The data supporting the findings of this manuscript are available from the corresponding authors upon reasonable request. For the crystal structure of the uL14–RsfS complex, the coordinates and structure factors were deposited in the Protein Data Bank, PDB 6SJ5 [10.2210/pdb6SJ5/pdb]. For the cryo-EM structure of the 50S–RsfS complex, coordinates were deposited in the Protein Data Bank, PDB 6SJ6 [10.2210/pdb6SJ6/pdb], while the cryo-EM map was deposited in the electron microscopy database, EMD-10212. The source data underlying Fig. 1a–c, e, f and Supplementary Fig. 1a, b, Supplementary Table 1 are provided as a Source Data file.

## Source Data

Source Data
