# Supplementary material for: Mechanism of ribosome shutdown by RsfS in Staphylococcus aureus revealed by integrative structural biology approach
Source: Nat Commun. 2020 Apr 3;11:1656. doi: 10.1038/s41467-020-15517-0 (PMC7125091; doi:10.1038/s41467-020-15517-0)
Supplement: Supplementary file 1 — SUPPLEMENTARY INFORMATION [file 41467_2020_15517_MOESM1_ESM.pdf]

**SUPPLEMENTARY INFORMATION**

# Mechanism of ribosome shutdown by RsfS in *Staphylococcus aureus* revealed by integrative structural biology approach

Khusainov et al.

Supplementary Figures 1 – 6

Supplementary Tables 1 – 3

# SUPPLEMENTARY FIGURES

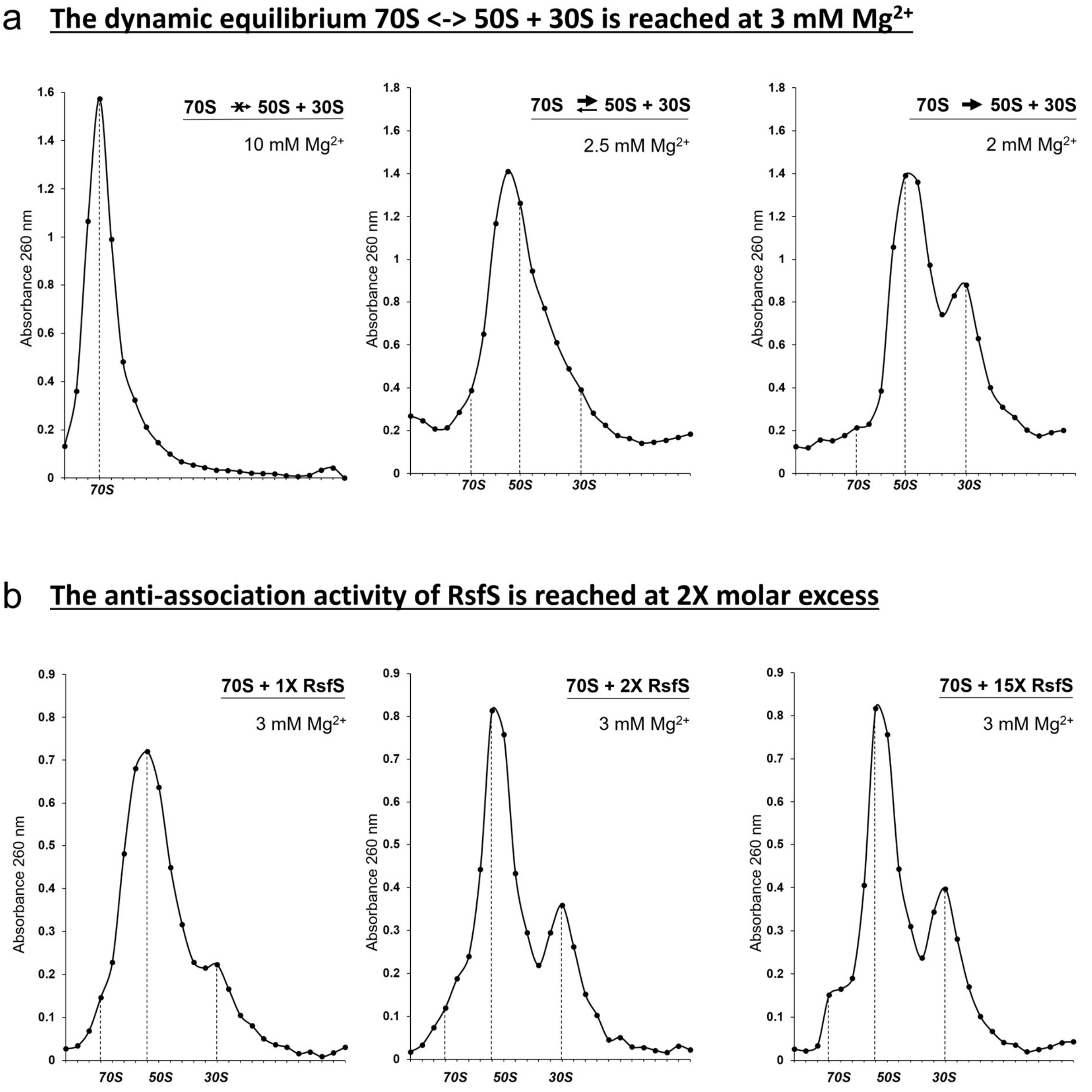


**Supplementary Figure 1.** Sucrose gradient (SG) profiles of the 70S and RsfS at semi-dissociation conditions. **a** Effect of magnesium on *S. aureus* ribosome subunits association. At 10 mM $Mg^{2+}$ the 70S ribosomes remain intact (left panel); at 2.5 mM $Mg^{2+}$ the ribosomes dissociate partly with the equilibrium shifted towards dissociation (middle panel); at 2 mM $Mg^{2+}$ 70S ribosomes are fully dissociated into 30S and 50S individual subunits (right panel). **b** RsfS titration to determine the 70S:RsfS ratio sufficient to prevent ribosome re-association at 3 mM $Mg^{2+}$. At 1:1 ratio, the equilibrium is partly shifted towards dissociation (left panel), the maximum activity reached at 1:2 ratio (middle panel). Increasing RsfS to 15X molar excess did not significantly affect the profile further (right panel). Source data for Fig 1a-b and are provided as a Source Data file.

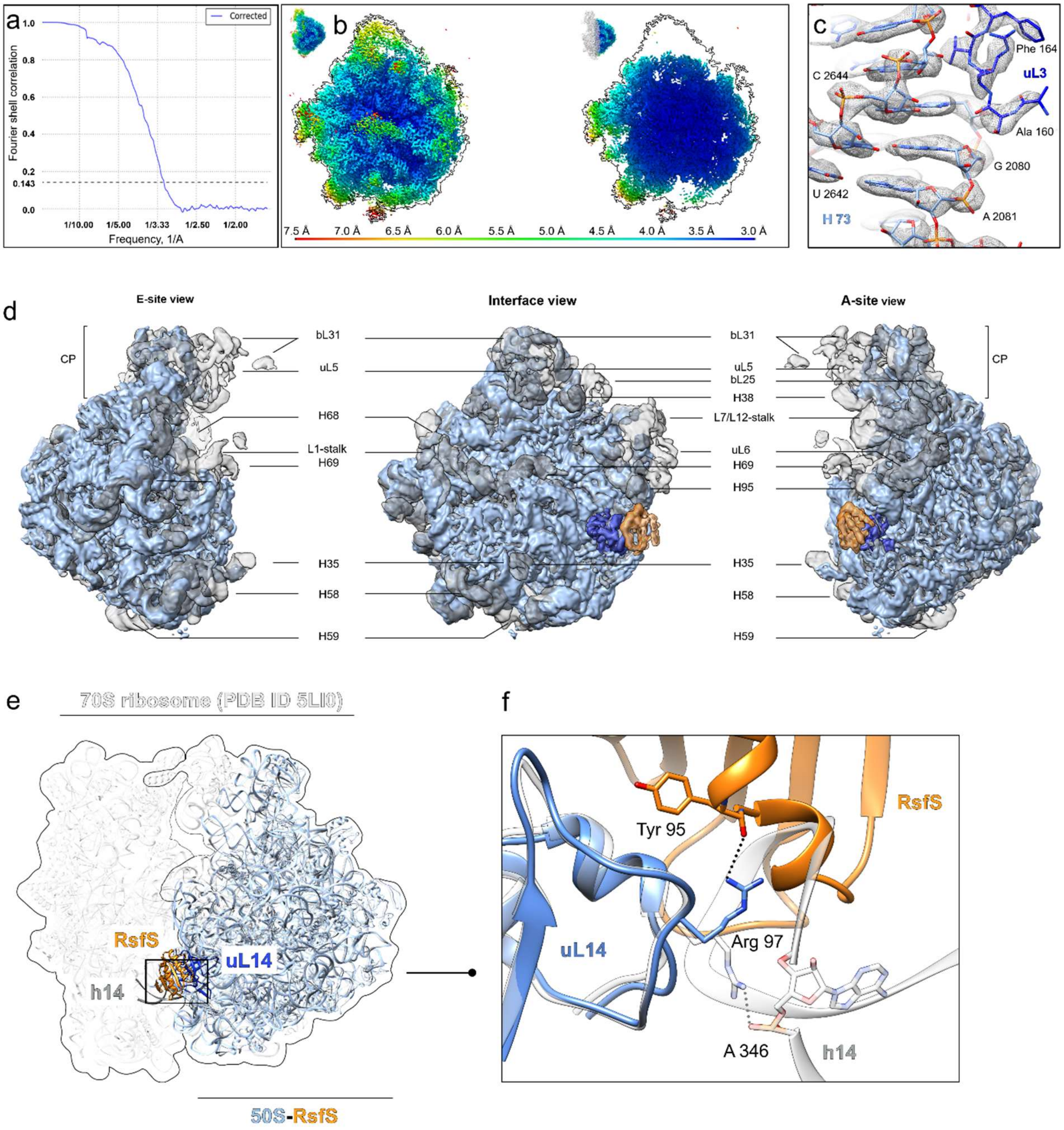


**Supplementary Figure 2.** Cryo-EM structure analysis of the 50S-RsfS complex. **a** Fourier Shell Correlation (FSC) curve indicating the overall resolution of the final 50S-RsfS reconstruction. **b** Local resolution (rainbow scale) displayed on representative slices of the density map for the 50S-RsfS complex. **c** The density and the model of 23S rRNA and uL3 protein in the core of the 50S. **d** Comparison of the cryo-EM density of the 50S-RsfS complex (current study, blue) and vacant 70S ribosome (PDB ID 5LI0 [http://dx.doi.org/10.2210/pdb5LI0/pdb][1], grey). Both maps were Gaussian filtered with the width equal to 3.5 pixel size of the initial maps. In the absence of small subunit and potentially due to additional purification steps in low $Mg^{2+}$ concentration, the periphery and the interface of the 50S became partly disordered or completely absent on the cryo-

EM map (regions have been labeled). Despite the overall disorder, uL14 (dark blue) and RsfS (orange) proteins had a high signal on the cryo-EM density map of the 50S-RsfS complex. For representation clarity, the density maps of the uL14 and RsfS were also coarsely segmented from the original map using Segger [2] and Gaussian filtered with the width equal to 3.5 pixel size and colored accordingly. CP – central protuberance. **e** An overlay of 50S-RsfS complex (blue ribbon) on the vacant *S. aureus* 70S (faint grey ribbon). The binding site of RsfS (orange) would clash with the helix 14 (h14) of the 16S rRNA (dark grey), thus preventing 30S association. **f** A close-up view of the clashing region, where helix 14 and loop Met27 – Asp35 from RsfS would impede rearrangement of the rRNA upon joining of the 30S. More into details, the side chain of Arg97 of uL14 is interacting with the backbone of A346 of the 16S rRNA in the presence of the 30S or with the backbone of Tyr95 in RsfS.

a **uL14**

```
S.aureus          MIQQETRLKVADNSGAREVLTIKVLGGSGRKTANIGDVIVCTVKNATPGGVVKKGDVVKA    60
B.subtilis        MIQQETRLKVADNSGAREVLTIKVLGGSGRKTANIGDVIVCTVKQATPGGVVKKGEVVKA    60
L.monocytogenes   MIQQESRMKVADNSGAREVLTIKVLGGSGRKTANIGDVVVCTVKQATPGGVVKKGEVVKA    60
E.faecalis        MIQQESRLRVADNSGAREILTIKVLGGSGRKTANIGDVIVATVKQATPGGVVKKGEVVKA    60
S.pneumoniae      MIQTETRLKVADNSGAREILTIKVLGGSGRKFANIGDVIVASVKQATPGGAVKKGDVVKA    60
S.iniae           MIQQETRLKVADNSGAREILTIKVLGGSGRKFANIGDVIVASVKQATPGGAVKKGDVVKA    60
A.flavithermus    MIQQETRMKVADNSGAREVLVIKVLGGSGRRYANVGDIVVATVKDATPGGVVKKGQVVKA    60
C.difficile       MIQQESRLRVADNSGAKEILCIRVLGGSKRRYGNIGDVIVATVKSATPGGVVKKGKVVKA    60
L.lactis          MIQTESRLKVADNSGAKELLTIRVLGGSSRKFAGIGDIVVATVKSAAPGGAVKKGEVVKA    60
M.tuberculosis    MIQQESRLKVADNTGAKEILCIRVLGGSSRRYAGIGDVIVATVKDAIPGGNVKRGDVVKA    60
H.influenzae      MIQEQTMLDVADNSGARSVMCIKVLGGSHRRYAAIGDIIKITVKEAIPRGKVKKGDVLKA    60
E.coli            MIQEQTMLNVADNSGARRVMCIKVLGGSHRRYAGVGDIIKITIKEAIPRGKVKKGDVLKA    60
T.thermophilus    MIQPQTYLEVADNTGARKIMCIRVLKGSNAKYATVGDVIVASVKEAIPRGAVKEGDVVKA    60
V.cholerae        MIQMQTMLDAADNSGARSVMCIKVLGGSHRRYAHVGDIIKVTVKEAIPRGKVKKGDVLKA    60
P.aeruginosa      MIQTQSMLDVADNSGARRVMCIKVLGGSHRRYAGIGDIIKVTVKEAIPRGKVKKGQVMTA    60

S.aureus          VIVRTKSGVRRNDGSYIKFDENACVIIRD-DKGPRGTRIFGPVARELREGNFMKIVSLAPEVL    122
B.subtilis        VIVRTKSGARRSDGSYISFDENACVIIRD-DKSPRGTRIFGPVARELRENNFMKIVSLAPEVI    122
L.monocytogenes   VIVRTKSGARRQDGSYIKFDENACVIIRD-DKSPRGTRIFGPVARELRENNFMKIVSLAPEVL    122
E.faecalis        VIVRTKSGARRADGSYIKFDENAAVIIRD-DKSPRGTRIFGPVARELRENNFMKIVSLAPEVL    122
S.pneumoniae      VIVRTKSGARRADGSYIKFDENAAVIIRE-DKTPRGTRIFGPVARELREGGFMKIVSLAPEVL    122
S.iniae           VIVRTKTGARRPDGSYIKFDDNAAVIIRD-DKTPRGTRIFGPVARELREGGYMKIVSLAPEVL    122
A.flavithermus    VVVRTKRGVRRTDGSYIRFDENACVIIRD-DKSPRGTRIFGPVARELREKDFMKIVSLAPEVI    122
C.difficile       VIVRTKQGMRRNDGSYISFDENAAVIIKD-DKTPVGTRIFGPVARELRDNEFMKIVSLAPEVL    122
L.lactis          VIVRTKSGAKRPDGSYIKFDENAAVLIRD-DKTPRGTRIFGPVARELREGGYMKIVSLAPEVL    122
M.tuberculosis    VVVRTVKERRRPDGSYIKFDENAAVIIKP-DNDPRGTRIFGPVGRELREKRFMKIISLAPEVL    122
H.influenzae      VVVRTKKGVRRPDGSVIRFDGNACVILNNNTEQPIGTRIFGPVTRELRSEKFMKIISLAPEVL    123
E.coli            VVVRTKKGVRRPDGSVIRFDGNACVLLNNNSEQPIGTRIFGPVTRELRSEKFMKIISLAPEVL    123
T.thermophilus    VVVRTKKEVKRPDGSAIRFDDNAAVIINN-QLEPRGTRVFGPVARELREKGFMKIVSLAPEVL    122
V.cholerae        VVVRTRKGVRRPDGSVIRFDRNACVLLNNNSEQPIGTRIFGPVTRELRNAKFMKIVSLAPEVL    123
P.aeruginosa      VVVRTKHGVRRTDGSIIRFDGNAAVLLNN-KQEPIGTRIFGPVTRELRTEKFMKIVSLAPEVL    122
```

b **RsfS**

```
S.aureus          ----MNSQELLAIAVDAIDNKKGEDTISLEMKGISDMTDYFVVTHGNNERQVQAIARAVKEVAN    60
A.flavithermus    ----MTERDILRIAVKAADEKKAENIVALNMKGISLVADYFMICHGNSDKQVQAIAREIKEKAE    60
B.subtilis        ----MNQKSILKIAAAACDDKRAEDILALDMEGISLVADYFLICHGNSDKQVQAIAREIKDQAD    60
L.monocytogenes   ----MNSYDTLMLTAKAADDKRAEDILALDMKGLSSFADYFVICHGNSDKQVQAIAREIKEKAL    60
E.faecalis        ---------MLEIAVKAADSKRAEEIVALDVREISLLADYFLICQANSERQINAIVDEINEQEA    55
S.iniae           ----MKKEELLDIVVKAADEKRAEDIIALNLEGLTSLTDYFVIATAGNTRQLEAIAENIREKVK    60
L.lactis          ----MDSKKLLEVVVKAADDKKALDIVALDMSEVTFVADYFVIMEAMNSRQLDAIADNIAEAAE    60
S.pneumoniae      ----MNEKELLELVVKAADEKRAEDILALDVQDLTSVTDYFVITSSMNSRQLDAIADNIREKVA    60
C.difficile       ----MTVEQMTKIAYDAIEDKLGQDTVIINIGKVSSLCDYFIITTASSQRQVKAIADNVEDELA    60
E.coli            ----MQGKALQDFVIDKIDDLKGQDIIALDVQGKSSITDCMIICTGTSSRHVMSIADHVVQESR    60
M.tuberculosis    MTANREAIDMARVAAGAAAAKLADDVVVIDVSGQLVITDCFVIASGSNERQVNAIVDEVEEKMR    64
P.aeruginosa      ----MQTEQLVQVAIDALEDLKAQDIVTLDVRDKTSVTDYMVIACGSSSRQVKSLADNVLTKAK    60
V.cholerae        ----MQGEALKDFLFDKADDMKAVDIVTLDVKEKSSVTDYMIVCTGTSKRHVASIAEHVANEAK    60
H.influenzae      -------MALVEFLMETLDGLKGTDIVHFDVRGKSSITDNMIICTGTSSRQVSAMADNLITECK    57
T.thermophilus    MVKAKEAVALIERIKELLAEKKAENVVALDLRRVSETLDYFVVASATSTPHLQALERHLLEKLE    64

S.aureus          EQNIE-VKRMEGYNEARWILIDLADVVVHVFHKDERNYYNIEKLYQDAPLESYGQVLA-----    117
A.flavithermus    EHDVV-VKRVEGFDEARWILVDLGDVVVHVFHKEDREYYNLERLWGDAPLEQIESDLRA----    118
B.subtilis        ENGIQ-VKKMEGFDEARWVLVDLGDVIVHVFHKDERSYYNLEKLWGDAPLADLDLGMNQ----    118
L.monocytogenes   ENQVD-VKRLEGFDAAKWILIDLGDVIIHVFHKEERSYYNLEKLWGDAPLVDVSAAFIS----    118
E.faecalis        KNQVE-VKRVEGKEGGKWILIDLGDVIVHVFQSSERAFYNLEKLWSDAPMVDLSAWVD-----    112
S.iniae           EAGGD-ASHVEGDSVTGWILLDLNDVVVHLFSEDERYHYNLEKLWHDAPEVALNLDLA-----    117
L.lactis          LAGAKAAGHIEGDAKTGWVLIDLGDIVVSVFGHDERGHFNLEKLWSDAPMVDISGFMAE----    119
S.pneumoniae      QAGFK-GSHIEGDATGGWVLLDLGAVVVHIFSEEMRAHYNLEKLWHEAHSVDLSETL------    116
C.difficile       KLGLE-PRGKEGQGTQTWVLLDYGDIMVHVFNEENRGFYNLEKLWKDAPYIDIDTLA------    116
E.coli            AAGLL-PLGVEGENSADWIVVDLGDVIVHVMQEESRRLYELEKLWS-----------------    105
M.tuberculosis    QAGYR-PARREGAREGRWTLLDYRDIVVHIQHQDDRNFYALDRLWGDCPVVPVDLSANSAGAQ    126
P.aeruginosa      ENGVK-PLGSEGLESGEWALLDLGDVVVHVMLPATRQFYDLERLWQGAEQSRAQHQPEE----    118
V.cholerae        LSGLQ-PLGMNGENEGEWVVLDMGSVMLHVMQEAPRELYQLEKLWG-----------------    105
H.influenzae      KAGFE-TFGEEGKNTADWIVVDLGQAIVHIMQRDAREMYQLEKLWA-----------------    102
T.thermophilus    EEDLR-PRPTAG-QSPRWVVLDYGEVVVHLMTPEAREYYDLEGFWADAERL------------    113
```

**Supplementary Figure 3**. Multiple sequence alignment of uL14 (panel **a**) and RsfS (panel **b**) proteins from 16 different bacterial species performed in Clustal Omega. Pink shading indicates positions that have a single, fully conserved residue (identity), yellow - conservation between amino acids with substantial chemical similarity and grey - amino acids with poor chemical similarity.

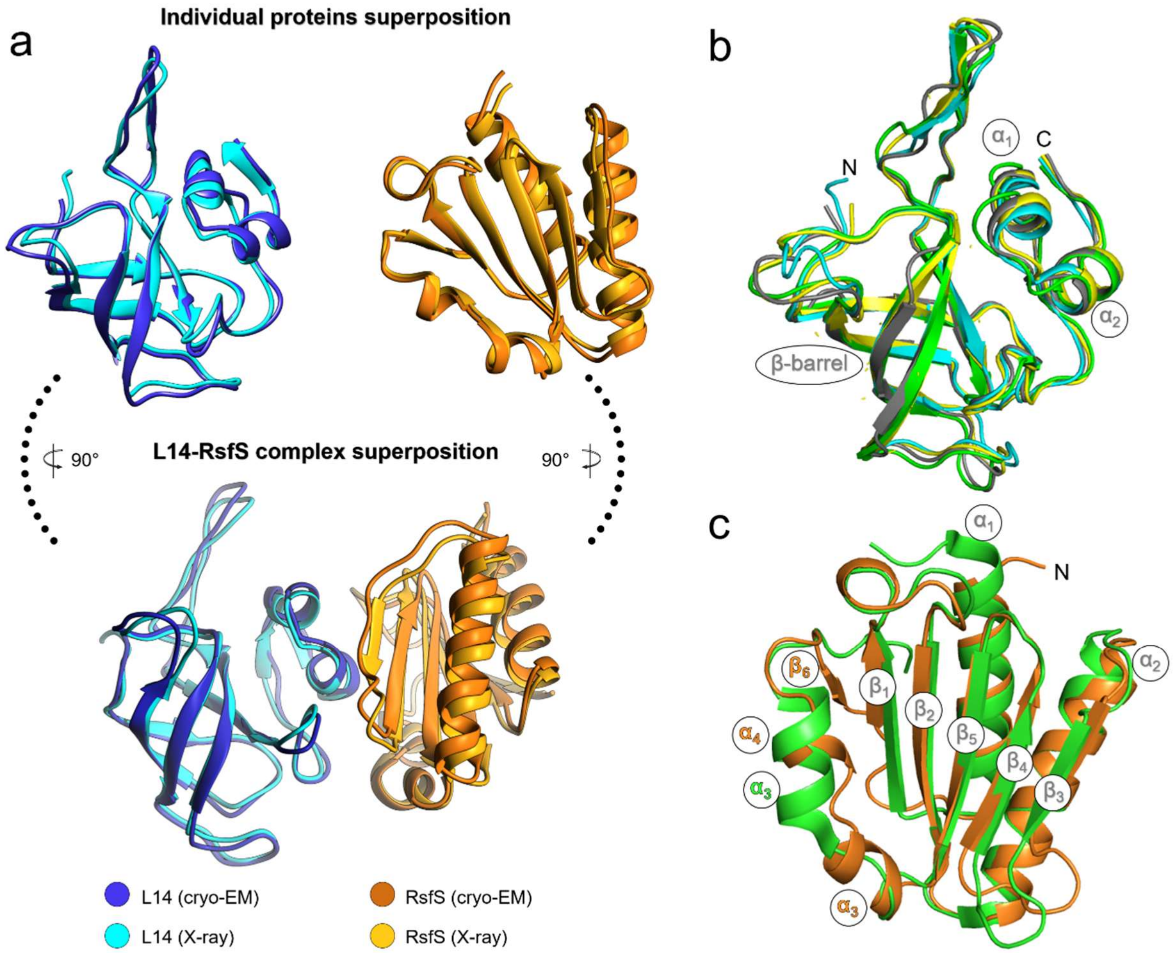


**Supplementary Figure 4.** Structural comparison of the uL14-RsfS complex within the 50S subunit solved by cryo-EM and crystal structure of the purified complex. **a** Superposition of individual proteins (upper part) and entire uL14-RsfS complex (lower part) derived from cryo-EM and the crystal structure. Color codes are indicated on the bottom of the figure panel. **b** Superposition of the crystal structure of uL14 from *S. aureus* (current study, cyan), *Geobacillus stearothermophilus* (PDB ID 1WHI [http://dx.doi.org/10.2210/pdb1WHI/pdb] [3], grey), *Thermus thermophilus* (PDB ID 6N9E [http://dx.doi.org/10.2210/pdb6N9E/pdb] [4], yellow) and *M. tuberculosis* (PDB ID 5V7Q [http://dx.doi.org/10.2210/pdb5V7Q/pdb] [5], green). c Superposition of *S. aureus* RsfS crystal structure (current study, orange) and RsfS from *M. tuberculosis* (PDB ID 4WCW [http://dx.doi.org/ 10.2210/pdb4WCW/pdb] [6], green). Grey labels of the secondary structure represent common elements, and colored labels represent species-specific features.

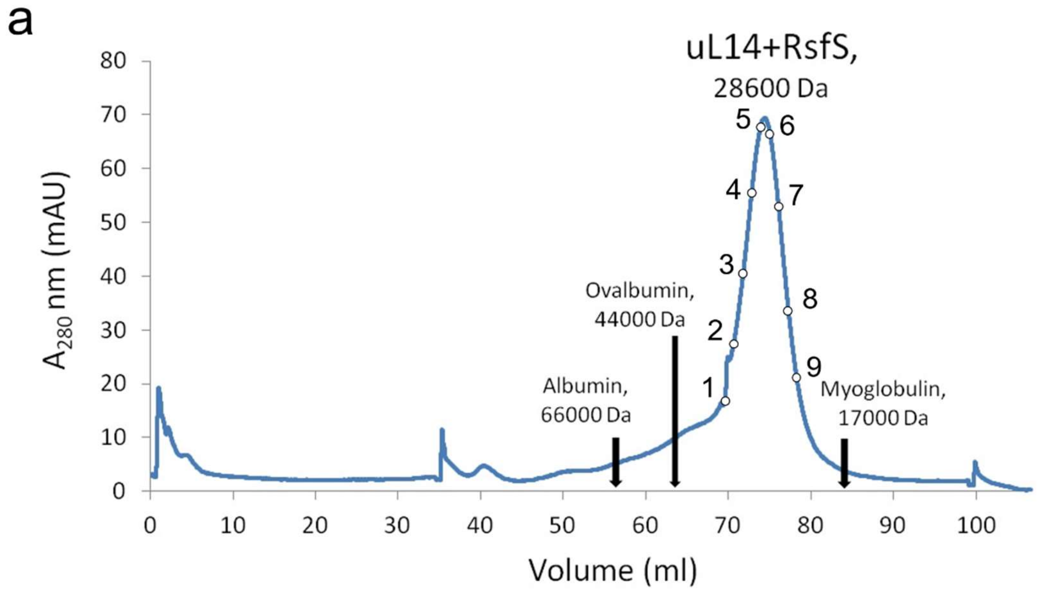


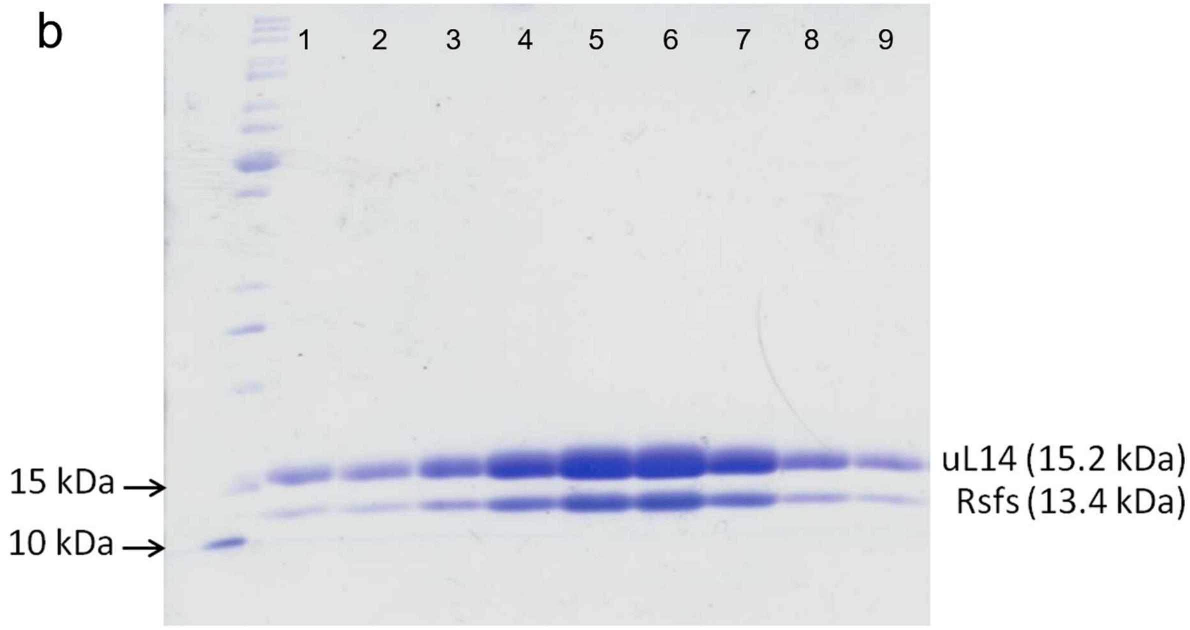


**Supplementary Figure 5.** Purification of the *S. aureus* uL14-RsfS complex for crystallization. **a** Size exclusion chromatography profile of the uL14-RsfS complex performed on a HiLoad 16/600 Superdex 75 prep grade column. Arrows indicate the positions of the molecular weight of reference proteins according to the manufacturer's calibration. The fractions that were taken for polyacrylamide gel electrophoresis (PAGE) are marked as white dots (1 to 9). **b** Analysis of the selected fractions by 15% PAGE. Fraction labels correspond to those in panel a).

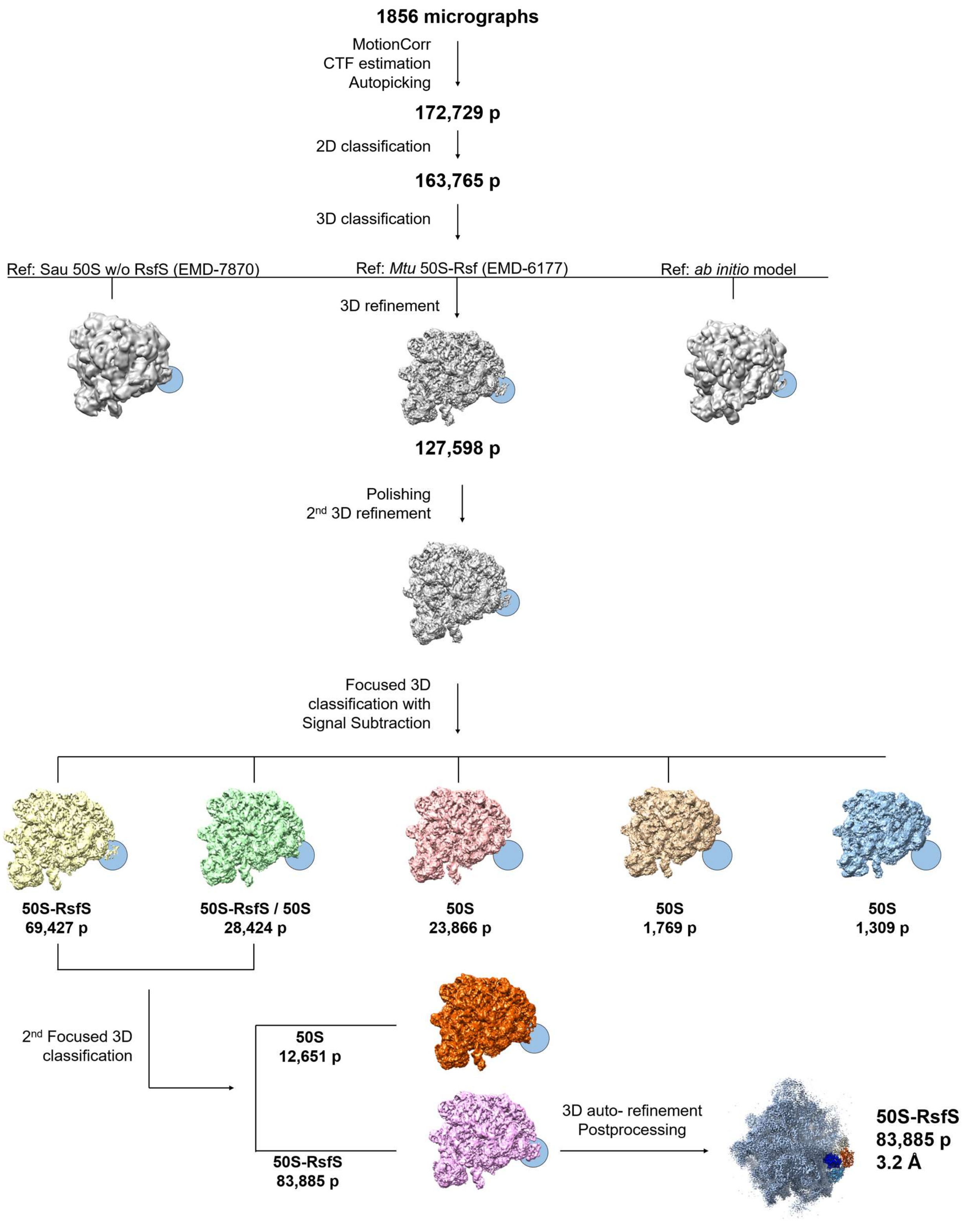


**Supplementary Figure 6**. Cryo-EM 3D particles sorting. The total number of particles (p) used for the 3D reconstructions is indicated below each structure. Blue circles point to the localization of RsfS on the 50S. Coloring of the final map is the same as in Fig. 2a.

# SUPPLEMENTARY TABLES

**Supplementary Table 1**. Mass spectrometry analysis of the 50S-RsfS complex used for cryo-EM structure determination.

| Accession | Gene | Description | Modifications | SAF |
|---|---|---|---|---|
| P0A0F8 | rplO | **50S ribosomal protein uL15** | Met-loss [N-Term] | 2.77 |
| P0A0G2 | rpmD | **50S ribosomal protein uL30** | Met-loss [N-Term] | 2.58 |
| Q2FXS8 | rplU | **50S ribosomal protein bL21** | Met-loss [N-Term] | 2.30 |
| P60430 | rplB | **50S ribosomal protein uL2** | | 1.89 |
| Q2FW38 | rplM | **50S ribosomal protein uL13** | Met-loss [N-Term] | 1.88 |
| Q2FW18 | rplE | **50S ribosomal protein uL5** | | 1.87 |
| Q2FW06 | rplC | **50S ribosomal protein uL3** | | 1.82 |
| Q2FW11 | rplV | **50S ribosomal protein uL22** | | 1.65 |
| Q2FW33 | rplQ | **50S ribosomal protein bL17** | | 1.53 |
| Q2FW08 | rplW | **50S ribosomal protein uL23** | | 1.33 |
| Q2G0P0 | rplA | **50S ribosomal protein uL1** | | 1.12 |
| Q2FW21 | rplF | **50S ribosomal protein uL6** | | 1.09 |
| Q2G298 | rsfS | **Ribosomal silencing factor RsfS** | | 1.07 |
| Q2FW14 | rpmC | **50S ribosomal protein uL29** | | 1.00 |
| Q2FW29 | rpmJ | **50S ribosomal protein bL36** | | 0.97 |
| Q2FZ60 | rpmB | **50S ribosomal protein bL28** | | 0.95 |
| Q2FZ42 | rplS | **50S ribosomal protein bL19** | | 0.87 |
| Q2FWD8 | rpmE2 | **50S ribosomal protein bL31 type B** | | 0.83 |
| Q2FW07 | rplD | **50S ribosomal protein uL4** | Met-loss [N-Term] | 0.77 |
| Q2FXQ1 | rplT | **50S ribosomal protein bL20** | | 0.75 |
| Q2FW22 | rplR | **50S ribosomal protein uL18** | | 0.71 |
| Q2FXT0 | rpmA | **50S ribosomal protein bL27** | | 0.66 |
| Q2G0S0 | rplY | **50S ribosomal protein bL25** | | 0.66 |
| Q2G0N9 | rplJ | **50S ribosomal protein uL10** | | 0.56 |
| Q2FW16 | rplN | **50S ribosomal protein uL14** | | 0.55 |
| Q2FW17 | rplX | **50S ribosomal protein uL24** | | 0.50 |
| Q2FY22 | rpmG2 | **50S ribosomal protein bL33 2** | | 0.39 |
| P0A0F4 | rplK | **50S ribosomal protein uL11** | | 0.39 |
| Q2FW31 | rpsK | 30S ribosomal protein uS11 | | 0.28 |
| Q2FW13 | rplP | **50S ribosomal protein uL16** | | 0.27 |
| Q2FXQ0 | rpmI | **50S ribosomal protein bL35** | | 0.24 |
| Q2FXZ7 | rpsU | 30S ribosomal protein bS21 | | 0.24 |
| Q2FW10 | rpsS | 30S ribosomal protein uS19 | | 0.21 |
| Q2G113 | rpsF | 30S ribosomal protein bS6 | | 0.18 |
| P48940 | rpsG | 30S ribosomal protein uS7 | | 0.17 |
| Q2FZ25 | rpsB | 30S ribosomal protein uS2 | | 0.16 |
| Q2FXK6 | rpsD | 30S ribosomal protein uS4 | | 0.13 |
| Q2FW12 | rpsC | 30S ribosomal protein uS3 | | 0.10 |
| Q2FW23 | rpsE | 30S ribosomal protein uS5 | | 0.10 |
| Q2FZ45 | rpsP | 30S ribosomal protein bS16 | | 0.08 |

| | | | | |
|---|---|---|---|---|
| Q2G2A4 | SAOUHSC_01042 | Dihydrolipoamide acetyltransferase component of pyruvate dehydrogenase complex | | 0.08 |
| Q2FXY2 | SAOUHSC_01698 | Uncharacterized protein | | 0.07 |
| Q2G2A5 | SAOUHSC_01041 | Pyruvate dehydrogenase complex, E1 component, pyruvate dehydrogenase beta subunit, putative | Met-loss [N-Term] | 0.07 |
| Q2FW15 | rpsQ | 30S ribosomal protein S17 | | |
| Q2FZG4 | SAOUHSC_01040 | Pyruvate dehydrogenase complex, E1 component, alpha subunit, putative | | 0.07 |
| Q2FW20 | rpsH | 30S ribosomal protein uS8 | Met-loss [N-Term] | 0.06 |
| P0A0H0 | rpsL | 30S ribosomal protein uS12 | Met-loss [N-Term] | 0.05 |
| Q2FW30 | rpsM | 30S ribosomal protein uS13 | | 0.04 |
| Q2FW39 | rpsI | 30S ribosomal protein uS9 | | 0.03 |
| P02976 | spa | Immunoglobulin G-binding protein A | | 0.02 |
| P0CE47 | tufA | Elongation factor Tu 1 E. coli (K12) | | 0.02 |

Protein identification was performed from liquid sample. Proteins ranking is based on SAF (Spectrum Abundance Factor), from the most abundant to the less abundant. Components of the 50S subunit r-proteins and RsfS are shown in bold.

**Supplementary Table 2.** Potential hydrogen bonds between uL14 and RsfS in the two heterodimers of the unit cell in the crystal structure.

| Contact atoms | | | | Distance (Å) | |
|---|---|---|---|---|---|
| uL14 | Conservation | RsfS | Conservation | Heterodimer 1 | Heterodimer 2 |
| Arg 107[ NH1] | *** | Met 33[ O ] | * | 2.89 | 2.91 |
| Arg 107[ O ] | *** | Arg 68[NH1] | | 3.01 | - |
| Asn 110[OD1] | | Arg 68[NH1] | | 3.25 | - |
| Met 112[ N ] | *** | Glu 70[ OE2] | | 2.57 | 2.88 |
| Lys 113[ N ] | *** | Glu 70[ OE1] | | 3.05 | 3.08 |
| Lys 113[ NZ ] | *** | Trp 77[ O ] | *** | 2.51 | 2.75 |
| Arg 107[ NH2] | *** | Asp 81[ OD1] | *** | 2.54 | 2.30 |
| Arg 107[ NE ] | *** | Asp 81[ OD2] | *** | 2.35 | 2.42 |
| Arg 107[ NH2] | *** | Ala83[ O ] | | 3.17 | 3.31 |
| Arg 97[ NH1] | *** | Tyr 98[ O ] | ** | 2.74 | 3.00 |
| Arg 97[ NH2] | *** | Tyr 98[ O ] | ** | 2.85 | 2.69 |
| Ser 116[ O ] | *** | Tyr 104[ OH ] | ** | 2.50 | 2.72 |
| Contacts mediated by water molecules | | | | | |
| Asp 90[ OD2 ] | | Trp 77[ N ] | *** | | |
| Pro 93 [ O ] | *** | Tyr 98[ OH ] | ** | | |

Asterisks define the conservation of these amino acids between 16 analyzed species presented in Supplementary Fig. 3 a,b (*** – identical (100%), ** – high conservative (>50%), * – low conservative (<50%), space – non-conserved amino acids).

**Supplementary Table 3.** Hydrophobic interactions (< 5 Å) between uL14 and RsfS in the two heterodimers of the unit cell in the crystal structure.

| Contact amino acids | | | | | |
|---|---|---|---|---|---|
| uL14 | Conservation | RsfS | Conservation | Heterodimer 1 | Heterodimer 2 |
| Met 112 | *** | | * | + | + |
| Val 115 | ** | Met 33 | | + | + |
| Val 121 | *** | | | + | + |
| Met 112 | *** | Phe 37 | ** | - | + |
| Pro 93 | *** | Trp 77 | *** | + | + |
| Leu 117 | *** | | | + | + |
| Met 112 | *** | Leu 79 | ** | + | + |
| | | Val 86 | ** | + | + |
| Pro 93 | *** | Tyr 98 | ** | - | + |
| Leu 117 | *** | | | + | + |
| Leu 117 | *** | Ile 100 | ** | + | + |
| Leu 117 | *** | Leu 103 | ** | + | + |

Asterisks define the conservation of these amino acids between 16 analyzed species presented in Supplementary Fig. 3 a,b (*** – identical (100%), ** – high conservative (>50%), * – low conservative (<50%), space – non-conserved amino acids).

# SUPPLEMENTARY REFERENCES


1. Khusainov, I. *et al.* Structure of the 70S ribosome from human pathogen Staphylococcus aureus. *Nucleic Acids Res.* **44**, gkw933 (2016).

2. Pintilie, G. D., Zhang, J., Goddard, T. D., Chiu, W. & Gossard, D. C. Quantitative analysis of cryo-EM density map segmentation by watershed and scale-space filtering, and fitting of structures by alignment to regions. *J. Struct. Biol.* **170**, 427–438 (2010).

3. Davies, C., White, S. W. & Ramakrishnan, V. The crystal structure of ribosomal protein L14 reveals an important organizational component of the translational apparatus. *Structure* **4**, 55–66 (1996).

4. Melnikov, S. V. *et al.* Mechanistic insights into the slow peptide bond formation with D-amino acids in the ribosomal active site. *Nucleic Acids Res.* (2019). doi:10.1093/nar/gky1211

5. Yang, K. *et al.* Structural insights into species-specific features of the ribosome from the human pathogen Mycobacterium tuberculosis. *Nucleic Acids Res.* **45**, 10884–10894 (2017).

6. Li, X. *et al.* Structure of Ribosomal Silencing Factor Bound to Mycobacterium tuberculosis Ribosome. *Structure* **23**, 1858–1865 (2015).
