# Supplementary material for: Mechanism of ribosome shutdown by RsfS in Staphylococcus aureus revealed by integrative structural biology approach
Source: Nat Commun. 2020 Apr 3;11:1656. doi: 10.1038/s41467-020-15517-0 (PMC7125091; doi:10.1038/s41467-020-15517-0)
Supplement: Supplementary file 2 — Peer Review [file 41467_2020_15517_MOESM2_ESM.pdf]

Reviewers' comments:

Reviewer #1 (Remarks to the Author):

This manuscript from Khusainov presents important work and has the potential to be of great interest to the readership of Nature Communications. However, in the present form the manuscript has important omissions in the way the data is presented and interpreted. I believe if the comments indicated below are considered, the manuscript quality will significantly improve and I could support his publication in Nature Communications.

The manuscript describes the cryo-EM structure of the 50S subunit in complex with the universally conserved ribosome silencing factor RsfS. The authors also obtained the crystal structure of RsfS in complex with r-protein uL14 which constitute the main interaction partner of the factor in the 50S+RsfS. These are my main concerns:

1. Fig. 1 describes first a number of experiments in which the effects of Mg^{2+} concentration is tested in the stability of the 70S ribosomes. No surprisingly, they found that lower Mg^{2+} concentration triggers dissociation of the 70S ribosomes. This is an important experiment for what is tested later in panels e and f. However, the effect of Mg^{2+} in the association of the two subunits of the ribosome has been known for over two decades now. Here, they show that S. aureus ribosomes behaved quite similarly to E. coli. Perhaps, it is appropriate to move panels a to d to the Suppl. Figures.

2. I am not following the rational to conclude from the experiment in Fig. 1 that RsfS is unable to dissociate the 70S ribosome, but once bound to 50S subunit, prevents its re-association with the 30S. For these experiments, the authors use a 5-molar excess of RsfS and at this ratio they see an effect in subunit dissociation at 3 mM Mg^{2+} but not at 10 mM Mg^{2+}. To claim that RsfS is unable to dissociate the 70S ribosome and rule this possibility, they should also include the same type of experiment but using a higher molar excess of RsfS (10-fold or 15 fold). Ruling out that RsfS cannot dissociate the 70S at any molar ration will make more appropriate to conclude that the factor prevents re-association of the subunits.

3. It would also have been a nice addition to the experiment in Fig 1e, 1f and 1g to present a silver staining SDS PAGE or western blot to show whether the RsfS factor comigrates in the gradients in the same fractions that the 50S subunit does. Even better, in the mass spec characterization of the 50S+RsfS peak in Fig 1g it would have been useful to also quantify the relative amount of the factor with respect to the constitutive r-proteins existing in the sample.

4. The data presented is also consistent with a completely alternative interpretation on what occurs to the 50S subunit upon binding of RsfS. The authors postulate that in the cryo-EM structure of the 50S+RsfS complex, the peripherical region of the 50S subunit and subunit interface become destabilized, including very large rRNA helices such as H68. Density for these regions and some of the r-proteins is fragmented in the cryo-EM map. They postulate that these regions are not well defined in the cryo-EM map due to the lack of association with the 30S subunit. Furthermore, they indicate that the 5S rRNA region became distorted due to the additional centrifugation cycle. I believe this is incorrect. There are examples in the literature of cryo-EM structures of purified 50S subunits, not associated with a 30S subunit, where all these ribosomal motifs are perfectly represented by density. Two articles come to mind (Nikolay et al. (2018) Molecular Cell 70, 881–893 and Jomaa et al. (2014) NAR 42:3419-3445). It is clear though that their cryo-EM map shows many structural motifs absent or with a fragmented density. In addition, mass spectrometry analysis data in table 1 indicates that many of the r-proteins in the 50S+RsfS complex are present in very low occupancy. For example, the level of uL16 is ~10-fold lower than uL15. This important finding is not even discussed in the manuscript and is completely ignored. Taking all these data together, is it possible that RsfS binding triggers a structural destabilization of the 50S subunit causing the loss of some r-proteins and perhaps unfolding of some rRNA helices in CP and peripherical regions? I found interesting the suggestion from

the authors that RsfS prevent reassociation of the 50S subunit with the 30S subunit when its binding site does not overlap or block any of the essential inter subunit bridges between both subunits. An alternative possibility is that binding cause a reversion of the mature 50S subunit structure to a somehow immature structure unable to associate with the 30S subunit. One clear experiment necessary to test this hypothesis is to obtain a cryo-EM map of mature 50S subunits purified with the same purification approach used for the 50S+RsfS complex.

5.Regardless of my point #4 the authors should correlate the obtained mass spectrometry data with the cryo-EM. Are proteins showing low occupancy in mass spectrometry only displaying lack a fragmented or lack of density in the cryo-EM map?

6. The authors claim atomic resolution in the title of the manuscript and also several times through the text. The cryo-EM structure is obtained at 3.2Å and the X-ray structure of RsfS-UL14 was obtained at 2.3Å. Neither of them is real atomic resolution. It will be more appropriate the simply state the obtained resolution or say "high-resolution".

7. The negative staining EM image in Fig 1h is unnecessary. If the purpose was to show that the purified complex obtained in Fig 1g was clean and suitable for cryo-EM, then the authors should display a cryo-EM field of view. This image would have given a good idea on the quality of the cryo-EM data that was used to produce the 3D reconstruction. Such a cryo-EM image should be place probably in the Suppl. Fig rather than in the main figures.

8. In Fig 2 and Suppl. Fig 2, authors should use colors that contrast better for RsfS and uL14. Blue and violet are not easily distinguished by a large group of color-blind people.

9. Fig 2G (line 634 should be g).

10. Fig 2 figure legend G speaks about a closed-up view but there is no close up view in the figure. Should be also 'g'.

11. Details on how the data was collected in F20 for negative staining are missing. What was the detector or CCD camera used? What was the total electron dose used? Was glow discharge applied to the grids?

12. Sequence alignments in Suppl. Fig 1 could be shown in more appealing manner. The authors should consider using some color to highlight important residues or areas of homology.

13. The FSC plot for the cryo-EM structure should be shown in the Suppl. Figures.

14. The authors use the map of Mycobacterium tuberculosis 50S ribosome in complex with Rsf (EMDB 6177) as an initial model both for class3d and autorefine steps of the processing pipeline. To ensure the obtained structure on the 50S+RsfS complex is not suffering from model biased, the authors should also try to use the structure of an empty 50S subunit. This will ensure that the extra density exhibited by the 50S+RsfS complex cryo-EM map is not imposed by the model.

15. The authors end up using about a 50% of the particle images collected to produce the cryo-EM map of the 50S+RsfS complex. It would be informative and useful to display in the suppl. Figures how the other classes looked like.

16. Line 308-309 Fig 1d should be Fig 1e and 1f.

17. Line 12 Fig 1e should be Fig 1g.

18. Use the new nomenclature for r-proteins in table 1 showing the mass spectrometry results.

19. There are many typos in the text, especially in the figure legends that should be revised and corrected.

Reviewer #2 (Remarks to the Author):

In the presented work by Khusainov et al., the authors obtained a high-resolution reconstruction of the large ribosomal subunit from Gram-positive bacterium S. aureus in complex with a hibernation factor RsfS. This structure together with biochemical data provides rational explanation of how RsfS works and significantly expands our fundamental understanding of how versatile the mechanisms of ribosome hibernation could be. In general, the process of ribosome hibernation itself attracts lots of attention recently, primarily because it plays pivotal role in survival of bacteria during various stresses, such as starvation, harsh conditions, or even treatment with antibiotics, and is thought to contribute to the development of drug resistance in some cases. Therefore, understanding the molecular mechanisms of ribosome hibernation is critical not only for our fundamental understanding of how the ribosome works but also for possible development of approaches to minimize pathogen survival during anti-microbial treatments. Although several mechanisms of ribosome hibernation (such as those operating through shortHPF/RMF, or YfiA, or longHPF protein factors) have been extensively studied, the described here RsfS-dependent mechanism is conceptually different in several aspects: (i) it operates on the level of individual large ribosomal subunits and acts as an anti-association factor; (ii) it does not cause ribosome dimerization and formation of the 100S dimers.

This work represents an excellent and exemplary study accomplished in the best traditions of the classic biochemistry and structural biology era! It is very rare these days to see a cryo-EM work, which is so nicely backed up by the biochemical data and high-resolution X-ray structure of the individual protein factor. The biochemical data provides insight into what RsfS factor is doing to the ribosome, while the structural part of the study explains how the antiassociation effect of RsfS is achieved. The cryo-EM structure of the 50S-RsfS complex is obtained at 3.2Å resolution, which is not very high resolution and, given that RsfS is binding at the periphery of the 50S subunit, the charge density is obviously not the best in that region (as can also be judged from the figures). I would like to emphasize this point because cryo-EM structures of the ribosome come out almost every week now and quite often far-reaching speculative and questionable conclusions are made based on the observation of just a few blobs in the charge density maps. Here, the situation and the quality of work is quite opposite – to ensure the proper and unambiguous fitting of the RsfS factor into the density, the authors obtained high-resolution X-ray structure of the factor complexed with its ribosome binding partner, the uL14 protein.

Overall, this is a well-executed and very interesting experimental work with important results and implications and definitely deserves to be published. The strong side of this work is the combination of methods that provide consistent results. The week side of this manuscript is a rather boring and monotonous description of all the alpha-helixes and beta-strands in the results section. The main findings of this study are within the scope of Nature Communications and, in my opinion, should be interesting not only to specialists but also to the broad readership of this journal. Moreover, the manuscript is very well-written and organized. It has simple and clear illustrations that are self-explanatory (I was able to get the main message and the key findings of this work by looking at the figures, even without reading the main text or figure legends). In summary, this reviewer is enthusiastically in favor of publishing this work. Also, there are several minor critical points, which the authors might wish to address:

Comments, suggestions, and questions to the authors:

1. Line 33 (Abstract): The term "universally conserved" should be used only when a particular protein

is conserved between the three kingdoms of life (bacteria, eukaryotes, and archaea). If the RsfS factor is conserved only between bacteria, mitochondria, and chloroplasts, some other term should be used instead.

2. Line 77 (Introduction): Remove the word "maximum".
3. Line 89 (Results): It seems that the "sensitive parameter" is misused. Maybe change the beginning of this sentence to read "Since association of bacterial ribosomes is most sensitive to the concentration of Mg ions, we titrated…"

4. Lines 119-141 (Results): As a reader I expected to see description of the RsfS binding site on the large ribosomal subunit right in this part of the results section. Providing the details of how the structure was obtained and refined is, of course, great but the most important result of this work – "Where RsfS binds on the 50S subunit?" – should be mentioned right here. Also, I would like to suggest to the authors to consider moving some of the details of model building to the materials and methods section of the manuscript.

5. Line 123 (Results): Change "…high-end Titan Krios microscope…" to "…Titan Krios 300K microscope…" Everybody knows that it definitely high-end and couldn't be higher.

6. Line 136 (Results): Perhaps, authors wished to refer to Figure 2G (not 2D)?

7. Lines 150-152 (Results): It would be more clear to reader if authors would add a simple phrase stating that such a low RMSD means that the two copies are essentially the same.

8. Lines 154-168 (Results): Extremely boring and lengthy description of the uL14-RsfS crystal structure. The manuscript would benefit from cutting this paragraph down to only a few sentences, in which the authors should describe the key findings of their crystal structure that are crucial to the rest of the story rather than monotonously describe the residues involved in the contacts. All unnecessary details regarding which residue is interacting with which other residues in the structure should be moved to the supplementary materials or removed. In this case, less is more!

9. Line 177 (Results): Space is missing in "the16S".

10. Line 191 (Results): Remove the word "easily".

11. Line 191 (Results): Remove the word "proper" to read "…and ensured reliable flexible fitting of RsfS…".

12. Line 264 (Discussion): What is "joiap"? If this is the protein name of the homolog from plastids, perhaps proper capitalization is needed?

13. Line 269 (Discussion): Change "this" to "the" to read "…when the latter becomes available…"

14. Lines 278-279: It would be better to emphasize that this is only one particular ribosome hibernation mechanism that operates in S. aureus.

15. Line 286 (Discussion): Change "multi" to "multi-drug".
16. Figure 2: Annotate on the figure what is the difference between panels A and B.

17. Figure 2, Panel E: Annotate that RsfS is shown.

18. Figure 3: It is unclear what is the purpose of showing electrostatic potential surface of the two proteins. Panels B and C appear to be somewhat redundant to panels D and H. Perhaps, the panel on the lower right should be labeled as H. Also, the black annotations of alpha-helixes and beta-strands

in panels B and C are not clearly visible. Using yellow color could help.

19. Tables 1, 2, and 3 should be moved to the Supplementary Materials section because they do not provide any data that is necessary and essential to the story. The data from these tables is important but only to a few specialists in the field.

We thank the reviewers for their valuable comments and suggestions. After addressing most of them, in our opinion the manuscript became more complete and structured. Below are the point-by-point responses.

Reviewers' comments:

Reviewer #1 (Remarks to the Author):

This manuscript from Khusainov presents important work and has the potential to be of great interest to the readership of Nature Communications. However, in the present form the manuscript has important omissions in the way the data is presented and interpreted. I believe if the comments indicated below are considered, the manuscript quality will significantly improve and I could support his publication in Nature Communications.

The manuscript describes the cryo-EM structure of the 50S subunit in complex with the universally conserved ribosome silencing factor RsfS. The authors also obtained the crystal structure of RsfS in complex with r-protein uL14 which constitute the main interaction partner of the factor in the 50S+RsfS. These are my main concerns:

1. Fig. 1 describes first a number of experiments in which the effects of Mg2+ concentration is tested in the stability of the 70S ribosomes. No surprisingly, they found that lower Mg2+ concentration triggers dissociation of the 70S ribosomes. This is an important experiment for what is tested later in panels e and f. However, the effect of Mg2+ in the association of the two subunits of the ribosome has been known for over two decades now. Here, they show that S. aureus ribosomes behaved quite similarly to E. coli. Perhaps, it is appropriate to move panels a to d to the Suppl. Figures.

In our work, finding the exact concentration of magnesium that leads to 70S-50S-30S dynamic equilibrium was an essential part of biochemical purification of the 50S-RsfS complex since we could directly assess protein activity via sucrose gradients profiles. At the same time, we agree with the reviewer that our experiments are consistent with *E. coli* and thus, most of the Mg^{2+} titration experiments are moved to Supplementary Fig. 1a in the revised manuscript.

2. I am not following the rational to conclude from the experiment in Fig. 1 that RsfS is unable to dissociate the 70S ribosome, but once bound to 50S subunit, prevents its re-association with the 30S. For these experiments, the authors use a 5-molar excess of RsfS and at this ratio they see an effect in subunit dissociation at 3 mM Mg2+ but not at 10 mM Mg2+. To claim that RsfS is unable to dissociate the 70S ribosome and rule this possibility, they should also include the same type of experiment but using a higher molar excess of RsfS (10-fold or 15 fold). Ruling out that RsfS cannot dissociate the 70S at any molar ration will make more appropriate to conclude that the factor prevents re-association of the subunits.

Thank you to the reviewer for the suggestion. We added the requested experiments into the manuscript. We now show that RsfS is unable to dissociate the ribosome at 10 mM Mg^{2+} even using an excess as high as 15 times, as shown in Fig. 1g.

3. It would also have been a nice addition to the experiment in Fig 1e, 1f and 1g to present a silver staining SDS PAGE or western blot to show whether the RsfS factor comigrates in the gradients in the same fractions that the 50S subunit does. Even better, in the mass spec characterization of the 50S+RsfS peak in Fig 1g it would have been useful to also quantify the relative amount of the factor with respect to the constitutive r-proteins existing in the sample.

> We agree with the reviewer and we thus performed the analysis of the ribosomal fractions using Western blot and showed the co-migration of the RsfS together with the 50S subunit fraction (Fig. 1d). Additionally, the mass spectrometry analysis that is already present in the paper was performed exactly to the 50S-RsfS peak (old version Fig. 1g) which is mentioned by the reviewer. It clearly shows the presence of RsfS in this fraction, and since our goal is not to quantify the 50S-RsfS complex formation, we think that such characterization might be enough for the scope of the manuscript. Moreover, the quantification of the 50S particles bound to RsfS was assessed by cryo-EM classification and sorting. We now added this information as Supplementary Figure 6.
> The detailed description of these and other new experiments is placed in the Methods section.

4. The data presented is also consistent with a completely alternative interpretation on what occurs to the 50S subunit upon binding of RsfS. The authors postulate that in the cryo-EM structure of the 50S+RsfS complex, the peripherical region of the 50S subunit and subunit interface become destabilized, including very large rRNA helices such as H68. Density for these regions and some of the r-proteins is fragmented in the cryo-EM map. They postulate that these regions are not well defined in the cryo-EM map due to the lack of association with the 30S subunit. Furthermore, they indicate that the 5S rRNA region became distorted due to the additional centrifugation cycle. I believe this is incorrect.

> We thank the reviewer for bringing up this idea. It made us think that we are missing one important experiment. Therefore, in order to clarify the effect of the RsfS binding on the integrity of the 50S subunit we first formed the 50S-RsfS complex at 3mM Mg^{2+} with the following increase of Mg^{2+} concentration to 10 mM. It showed that the 50S-RsfS + 30S mixture is able to convert back into 70S + RsfS once Mg^{2+} is increased. Thus, this would not support the hypothesis that RsfS binding triggers the degradation of the 50S, at least *in vitro*. Additionally, it confirmed the pivotal role of Mg^{2+} ions for the 70S association even in the presence of anti-association factors. In the main text we rephrased this part and highlight the role of Mg^{2+} in destabilisation.

There are examples in the literature of cryo-EM structures of purified 50S subunits, not associated with a 30S subunit, where all these ribosomal motifs are perfectly represented by density. Two articles come to mind (Nikolay et al. (2018) Molecular Cell 70, 881–893 and Jomaa et al. (2014) NAR 42:3419-3445).

> We agree with the reviewer that the structural stability of the 50S should not be caused only by the absence of the 30S. In turn, we propose that all these distortions may be caused by a multitude of factors: i) we work with wild type *S. aureus* cells which contain all natural RNases; ii) we keep 3 mM Mg^{2+} concentration for complex formation and additionally perform centrifugation in such conditions, known to affect stability of the ribosome; iii) in total 4 high-speed centrifugations were used (2 during 70S purification + 2 during 50S-RsfS purification); iv) the ribosome from *S. aureus* seem to be generally less stable than *E. coli* MRE600 or similar RNase-free strains.
>
> Currently, there is only one 50S cryo-EM structure from *S. aureus* available (Eyal et al., unpublished, EMD-0243) with similarly weaker resolved H38, H68 and components of CP including 5S rRNA, consistent with our study. However, no experimental information is available for this study, so we can't do a direct comparison of sample preparation.

Regarding the biogenesis studies, the sample preparation for cryo-EM analysis was performed using an increased concentration of magnesium (15 mM or 20 mM for Jomaa et al and Nikolay et al respectively), which is a common approach when studying ribosome biogenesis in bacteria. We believe that high Mg^{2+} concentration is critical to keep the integrity of individual subunits in these cases.

Taking all this into account, there are several factors influencing the stability of the *S. aureus* 50S and we would like to avoid any speculation on the destabilizing role of RsfS for 50S subunits without having supporting evidence for this.

It is clear though that their cryo-EM map shows many structural motifs absent or with a fragmented density. In addition, mass spectrometry analysis data in table 1 indicates that many of the r-proteins in the 50S+RsfS complex are present in very low occupancy. For example, the level of uL16 is ~10-fold lower than uL15. This important finding is not even discussed in the manuscript and is completely ignored.

We believe that such inconsistency is just a matter of technical approach and the complexity of the sample. For the proteins with the highest score, the most detected peptides were those exposed to the surface. For example, in protein uL15, FSASAAEAIDAK sequence has the highest number peptide-spectrum match (PSM). This sequence belongs to a flexible surface-oriented region at the C-terminal part of the protein. In contrast, the protein uL16 is strongly associated with 23S rRNA and its detection by MS is limited. This is quite a common issue in the MS analysis of liquid ribosome samples. To properly quantify all r-proteins the quantitative MS would be preferable and a thorough sample preparation procedure would have to be exploited. Knowing these methodological aspects, we used MS analysis to verify the presence of RsfS and 50S r-proteins in the sample. Directly correlating the cryo-EM density signal with the MS detection would be *a priori* error-prone.

The raw MS data is now submitted as a Supplementary data file 1.

Taking all these data together, is it possible that RsfS binding triggers a structural destabilization of the 50S subunit causing the loss of some r-proteins and perhaps unfolding of some rRNA helices in CP and peripherical regions?

I found interesting the suggestion from the authors that RsfS prevent reassociation of the 50S subunit with the 30S subunit when its binding site does not overlap or block any of the essential inter subunit bridges between both subunits. An alternative possibility is that binding cause a reversion of the mature 50S subunit structure to a somehow immature structure unable to associate with the 30S subunit. One clear experiment necessary to test this hypothesis is to obtain a cryo-EM map of mature 50S subunits purified with the same purification approach used for the 50S+RsfS complex.

We cannot fully rule out such a scenario *in vivo*; however, our experiments show that this is very unlikely to occur *in vitro*. To provide further evidence in support of this, we compared the classes of the 50S bound and not bound to RsfS side by side, as output from 3D classification (see figure below). The distortions of the 50S particles are very similar.

We hope that all our answers convinced the reviewer that addressing this particular question requires separate complex research and was not the aim of this manuscript, but we really appreciated such comments and we think this might deserve a more thorough investigation in the future.

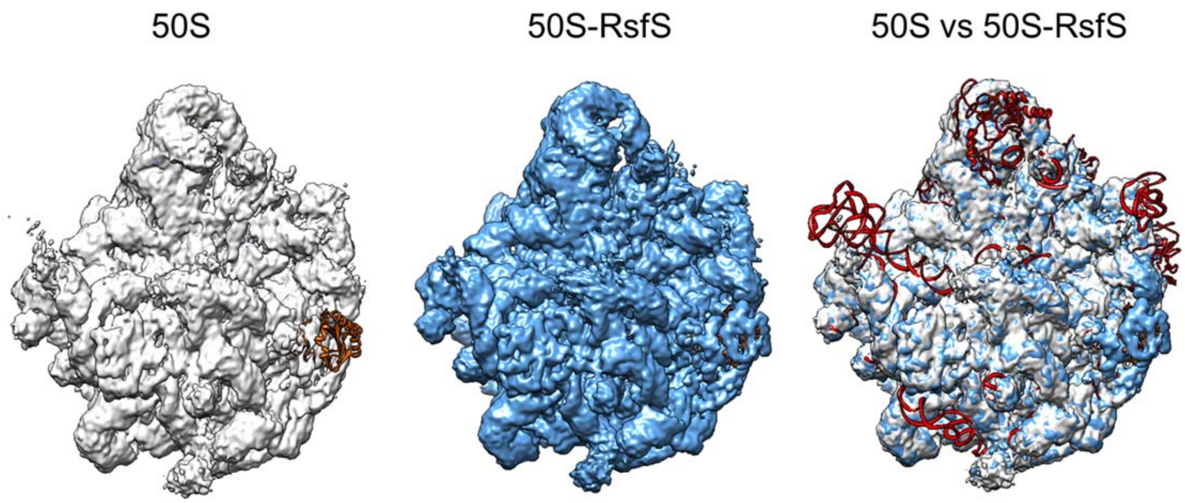

5.Regardless of my point #4 the authors should correlate the obtained mass spectrometry data with the cryo-EM. Are proteins showing low occupancy in mass spectrometry only displaying lack a fragmented or lack of density in the cryo-EM map?

Please refer to the previous point #4, in which this question has been addressed.

6. The authors claim atomic resolution in the title of the manuscript and also several times through the text. The cryo-EM structure is obtained at 3.2Å and the X-ray structure of RsfS-UL14 was obtained at 2.3Å. Neither of them is real atomic resolution. It will be more appropriate the simply state the obtained resolution or say "high-resolution".

We agree with the reviewer and changed these throughout the manuscript and in the title.

7. The negative staining EM image in Fig 1h is unnecessary. If the purpose was to show that the purified complex obtained in Fig 1g was clean and suitable for cryo-EM, then the authors should display a cryo-EM field of view. This image would have given a good idea on the quality of the cryo-EM data that was used to produce the 3D reconstruction. Such a cryo-EM image should be place probably in the Suppl. Fig rather than in the main figures.

We've replaced the negative staining EM image by the cryo-EM field of view (Fig. 1h).

8. In Fig 2 and Suppl. Fig 2, authors should use colors that contrast better for RsfS and uL14. Blue and violet are not easily distinguished by a large group of color-blind people.

We've changed the coloring in all the figures.

11. Details on how the data was collected in F20 for negative staining are missing. What was the detector or CCD camera used? What was the total electron dose used? Was glow discharge applied to the grids?

We have added this missing information to the respective Methods section.

12. Sequence alignments in Suppl. Fig 1 could be shown in more appealing manner. The authors should consider using some color to highlight important residues or areas of homology.

Corrected. The sequence alignment figure has now become Supplementary Figure 3.

13. The FSC plot for the cryo-EM structure should be shown in the Suppl. Figures.

Added as Supplementary Fig. 2a

14. The authors use the map of Mycobacterium tuberculosis 50S ribosome in complex with Rsf (EMDB 6177) as an initial model both for class3d and autorefine steps of the processing pipeline. To ensure the obtained structure on the 50S+RsfS complex is not suffering from model biased, the authors should also try to use the structure of an empty 50S subunit. This will ensure that the extra density exhibited by the 50S+RsfS complex cryo-EM map is not imposed by the model.

Thank you to this reviewer for the comment. To rule out the possibility that our 50S-RsfS reconstruction could be affected by reference bias, we used particles extracted after 2D classification to create an *ab initio* model (as explained in the Methods section) that does not contain any external bias. We did try also to use as a reference the *S. aureus* 50S cryo-EM density map (EMD-7870) that does not contain RsfS to run 3D classification and 3D refinement. This 3D classification also resulted in a reconstruction which contains RsfS protein. We added this in Supplementary figure 6 and amended the Methods section accordingly.

15. The authors end up using about a 50% of the particle images collected to produce the cryo-EM map of the 50S+RsfS complex. It would be informative and useful to display in the suppl. Figures how the other classes looked like.

We've added the description of the 50S-RsfS complex *in silico* sorting as Supplementary Fig. 6

18. Use the new nomenclature for r-proteins in table 1 showing the mass spectrometry results.

Corrected

9. Fig 2G (line 634 should be g).
10. Fig 2 figure legend G speaks about a closed-up view but there is no close up view in the figure. Should be also 'g'.
16. Line 308-309 Fig 1d should be Fig 1e and 1f.
17. Line 12 Fig 1e should be Fig 1g.
19. There are many typos in the text, especially in the figure legends that should be revised and corrected.

We now reshuffled the figures and corrected the main text and figure legends. We apologize for the appearance of typos and other inconsistencies in the original version.

Reviewer #2 (Remarks to the Author):

In the presented work by Khusainov et al., the authors obtained a high-resolution reconstruction of the large ribosomal subunit from Gram-positive bacterium S. aureus in complex with a hibernation factor RsfS. This structure together with biochemical data provides rational explanation of how RsfS works and

significantly expands our fundamental understanding of how versatile the mechanisms of ribosome hibernation could be. In general, the process of ribosome hibernation itself attracts lots of attention recently, primarily because it plays pivotal role in survival of bacteria during various stresses, such as starvation, harsh conditions, or even treatment with antibiotics, and is thought to contribute to the development of drug resistance in some cases. Therefore, understanding the molecular mechanisms of ribosome hibernation is critical not only for our fundamental understanding of how the ribosome works but also for possible development of approaches to minimize pathogen survival

during anti-microbial treatments. Although several mechanisms of ribosome hibernation (such as those operating through shortHPF/RMF, or YfiA, or longHPF protein factors) have been extensively studied, the described here RsfS-dependent mechanism is conceptually different in several aspects: (i) it operates on the level of individual large ribosomal subunits and acts as an anti-association factor; (ii) it does not cause ribosome dimerization and formation of the 100S dimers.

This work represents an excellent and exemplary study accomplished in the best traditions of the classic biochemistry and structural biology era! It is very rare these days to see a cryo-EM work, which is so nicely backed up by the biochemical data and high-resolution X-ray structure of the individual protein factor. The biochemical data provides insight into what RsfS factor is doing to the ribosome, while the structural part of the study explains how the antiassociation effect of RsfS is achieved. The cryo-EM structure of the 50S-RsfS complex is obtained at 3.2Å resolution, which is not very high resolution and, given that RsfS is binding at the periphery of the 50S subunit, the charge density is obviously not the best in that region (as can also be judged from the figures). I would like to emphasize this point because cryo-EM structures of the ribosome come out almost every week now and quite often far-reaching speculative and questionable conclusions are made based on the observation of just a few blobs in the charge density maps. Here, the situation and the quality of work is quite opposite – to ensure the proper and unambiguous fitting of the RsfS factor into the density, the authors obtained high-resolution X-ray structure of the factor complexed with its ribosome binding partner, the uL14 protein.

Overall, this is a well-executed and very interesting experimental work with important results and implications and definitely deserves to be published. The strong side of this work is the combination of methods that provide consistent results. The week side of this manuscript is a rather boring and monotonous description of all the alpha-helixes and beta-strands in the results section. The main findings of this study are within the scope of Nature Communications and, in my opinion, should be interesting not only to specialists but also to the broad readership of this journal. Moreover, the manuscript is very well-written and organized. It has simple and clear illustrations that are self-explanatory (I was able to get the main message and the key findings of this work by looking at the figures, even without reading the main text or figure legends). In summary, this reviewer is enthusiastically in favor of publishing this work. Also, there are several minor critical points, which the authors might wish to address:

Comments, suggestions, and questions to the authors:

1. Line 33 (Abstract): The term “universally conserved” should be used only when a particular protein is conserved between the three kingdoms of life (bacteria, eukaryotes, and archaea). If the RsfS factor is conserved only between bacteria, mitochondria, and chloroplasts, some other term should be used instead.

> Thank you for the comments. We amended the abstract according to the reviewer’s suggestion, abstract consistency and journal requirements. Now it states “conserved bacterial factor” in the abstract, while the presence of RsfS homologs in chloroplasts and mitochondria is mentioned later in the text, in the “Introduction” and “Discussion” sections.

2. Line 77 (Introduction): Remove the word “maximum”.

> Corrected

3. Line 89 (Results): It seems that the “sensitive parameter” is misused. Maybe change the beginning of this sentence to read “Since association of bacterial ribosomes is most sensitive to the concentration of Mg ions, we titrated…”

> Following the reviewer’s comment, we amended the sentence:
> “Since the association of bacterial ribosomes is most sensitive to the concentration of magnesium ions, we titrated Mg^{2+} while keeping the concentration of monovalent salts constant (Fig. 1a, Supplementary Fig. 1a).”

4. Lines 119-141 (Results): As a reader I expected to see description of the RsfS binding site on the large ribosomal subunit right in this part of the results section. Providing the details of how the structure was obtained and refined is, of course, great but the most important result of this work – “Where RsfS binds on the 50S subunit?” – should be mentioned right here. Also, I would like to suggest to the authors to consider moving some of the details of model building to the materials and methods section of the manuscript.

> Thank you to the reviewer for the comment. We have expanded the text (lines 136-142) and figures (Fig 2f) with the description of the RsfS binding cavity within the 50S.
>
> The details of model building were moved to the Methods section.

5. Line 123 (Results): Change “…high-end Titan Krios microscope…” to “…Titan Krios 300K microscope…” Everybody knows that it definitely high-end and couldn't be higher.

> Corrected

6. Line 136 (Results): Perhaps, authors wished to refer to Figure 2G (not 2D)?

> Corrected

7. Lines 150-152 (Results): It would be more clear to reader if authors would add a simple phrase stating that such a low RMSD means that the two copies are essentially the same.

> Corrected

8. Lines 154-168 (Results): Extremely boring and lengthy description of the uL14-RsfS crystal structure. The manuscript would benefit from cutting this paragraph down to only a few sentences, in which the authors should describe the key findings of their crystal structure that are crucial to the rest of the story rather than monotonously describe the residues involved in the contacts. All unnecessary details regarding

which residue is interacting with which other residues in the structure should be moved to the supplementary materials or removed. In this case, less is more!

> Following the reviewer's comment, we moved all the methodological information into the Methods section and focused exclusively on the key findings of the crystal structure. Additionally, we have merged this part with the comparative analysis of the cryo-EM 50S-RsfS and the X-ray structure of uL14-RsfS complex.

9. Line 177 (Results): Space is missing in "the16S".

> Corrected

10. Line 191 (Results): Remove the word "easily".

> Corrected

11. Line 191 (Results): Remove the word "proper" to read "…and ensured reliable flexible fitting of RsfS…".

> Corrected

12. Line 264 (Discussion): What is "joiap"? If this is the protein name of the homolog from plastids, perhaps proper capitalization is needed?

> Corrected

13. Line 269 (Discussion): Change "this" to "the" to read "…when the latter becomes available…"

> Corrected

14. Lines 278-279: It would be better to emphasize that this is only one particular ribosome hibernation mechanism that operates in S. aureus.

> Corrected

15. Line 286 (Discussion): Change "multi" to "multi-drug".

> Corrected

16. Figure 2: Annotate on the figure what is the difference between panels A and B.

> Corrected

17. Figure 2, Panel E: Annotate that RsfS is shown.

> Corrected

18. Figure 3: It is unclear what is the purpose of showing electrostatic potential surface of the two proteins. Panels B and C appear to be somewhat redundant to panels D and H. Perhaps, the panel on the lower right should be labeled as H. Also, the black annotations of alpha-helixes and beta-strands in panels B and C are not clearly visible. Using yellow color could help.

> We show electrostatic potential to display the general mode of interaction of RsfS with its primary binding partner uL14. We now moved this panel to Figure 2, which provides an overview of the RsfS interaction with the ribosome and displays the main structural elements both proteins that mediate this interaction. Thus, Figure 3 was re-organized and now only focuses on

the detailed contacts between uL14 and RsfS determined by the crystal structure. Annotations were changed in line with the other figures of the manuscript. Thank you.

19. Tables 1, 2, and 3 should be moved to the Supplementary Materials section because they do not provide any data that is necessary and essential to the story. The data from these tables is important but only to a few specialists in the field.

Corrected

REVIEWERS' COMMENTS:

Reviewer #1 (Remarks to the Author):

The authors did a thorough job in revising this manuscript and addressing all the concerns from this reviewer. I appreciate the additional experiments performed to solidify some of the conclusions.

Only one minor correction to be done. That will be to add a scale bar in fig. 1H.

Other than that, I believe the manuscript can now be published in Nature Communications.

REVIEWERS' COMMENTS:

Reviewer #1 (Remarks to the Author):

The authors did a thorough job in revising this manuscript and addressing all the concerns from this reviewer. I appreciate the additional experiments performed to solidify some of the conclusions.
Only one minor correction to be done. That will be to add a scale bar in fig. 1H.
Other than that, I believe the manuscript can now be published in Nature Communications.

We kindly thank the reviewer for the positive feedback on the revised manuscript. As suggested, we’ve added the scale bar to the electron micrograph image
